# Individualized prediction of three- and six-year outcomes of psychosis in a longitudinal multicenter study: a machine learning approach

**DOI:** 10.1038/s41537-021-00162-3

**Published:** 2021-07-02

**Authors:** Jessica de Nijs, Thijs J. Burger, Ronald J. Janssen, Seyed Mostafa Kia, Daniël P. J. van Opstal, Mariken B. de Koning, Lieuwe de Haan, GROUP investigators, Behrooz Z. Alizadeh, Agna A. Bartels-Velthuis, Nico J. van Beveren, Richard Bruggeman, Lieuwe de Haan, Philippe Delespaul, Jurjen J. Luykx, Inez Myin-Germeys, Rene S. Kahn, Frederike Schirmbeck, Claudia J. P. Simons, Therese van Amelsvoort, Jim van Os, Ruud van Winkel, Wiepke Cahn, Hugo G. Schnack

**Affiliations:** 1 Department of Psychiatry, University Medical Center Utrecht, UMC Utrecht Brain Center, Utrecht University, Utrecht, The Netherlands; 2 Arkin, Institute for Mental Health, Amsterdam, The Netherlands; 3 Department of Psychiatry, Amsterdam UMC, University of Amsterdam, Amsterdam, The Netherlands; 4 Altrecht, General Mental Health Care, Utrecht, The Netherlands; 5 University of Groningen, University Medical Center Groningen, University Center for Psychiatry, Rob Giel Research Center, Groningen, The Netherlands; 6 Department of Epidemiology, University Medical Center Groningen, Groningen, The Netherlands; 7 Antes Center for Mental Health Care, Rotterdam, the Netherlands; 8 Department of Neuroscience, Erasmus MC, Rotterdam, the Netherlands; 9 Department of Clinical and Developmental Neuropsychology, University of Groningen, Groningen, The Netherlands; 10 Department of Psychiatry and Psychology, School for Mental Health and Neuroscience, Maastricht University Medical Center, Maastricht, the Netherlands; 11 Department of Translational Neuroscience, University Medical Center Utrecht, UMC Utrecht Brain Center, Utrecht, The Netherlands; 12 Department of Neuroscience, Research Group Psychiatry, Center for Contextual Psychiatry, KU Leuven, Leuven, Belgium; 13 Department of Psychiatry, Icahn School of Medicine at Mount Sinai, New York, NY USA; 14 GGzE, Institute for Mental Health Care Eindhoven and De Kempen, Eindhoven, the Netherlands; 15 Department of Psychosis Studies, Institute of Psychiatry, King’s College London, King’s Health Partners, London, UK

**Keywords:** Psychosis, Schizophrenia

## Abstract

Schizophrenia and related disorders have heterogeneous outcomes. Individualized prediction of long-term outcomes may be helpful in improving treatment decisions. Utilizing extensive baseline data of 523 patients with a psychotic disorder and variable illness duration, we predicted symptomatic and global outcomes at 3-year and 6-year follow-ups. We classified outcomes as (1) symptomatic: in remission or not in remission, and (2) global outcome, using the Global Assessment of Functioning (GAF) scale, divided into good (GAF ≥ 65) and poor (GAF < 65). Aiming for a robust and interpretable prediction model, we employed a linear support vector machine and recursive feature elimination within a nested cross-validation design to obtain a lean set of predictors. Generalization to out-of-study samples was estimated using leave-one-site-out cross-validation. Prediction accuracies were above chance and ranged from 62.2% to 64.7% (symptomatic outcome), and 63.5–67.6% (global outcome). Leave-one-site-out cross-validation demonstrated the robustness of our models, with a minor drop in predictive accuracies of 2.3% on average. Important predictors included GAF scores, psychotic symptoms, quality of life, antipsychotics use, psychosocial needs, and depressive symptoms. These robust, albeit modestly accurate, long-term prognostic predictions based on lean predictor sets indicate the potential of machine learning models complementing clinical judgment and decision-making. Future model development may benefit from studies scoping patient’s and clinicians' needs in prognostication.

## Introduction

Schizophrenia is a heterogeneous illness and its long-term outcomes are highly variable^1–3^. Attempts to provide prognostic markers for long-term outcomes, such as Rumke’s “praecox feeling”, have appeared throughout medical history^4^, but despite an abundance of outcome predictors at group-level, such as sociodemographic characteristics, clinical markers, and neurocognitive markers^5,6^, at a patient-level, no valid prediction model for long-term outcome of schizophrenia is available to clinicians at present^7^. An additional challenge is that “outcome” entails symptomatic, social, functional, and personal dimensions, which are only partly interrelated^8,9^, and may have differing significance for individual patients^10,11^. These matters complicate clinical decision-making, for example when considering an early switch to clozapine^12^, antipsychotic dose reduction or discontinuation strategies^13^, allocations of sheltered housing^14^, or occupational support^15^. From a public health perspective, reliable long-term outcome prediction and the resulting treatment stratification are important, as demands usually outweigh the capacity of mental health institutions, even in countries with high mental healthcare expenses^16^.

Machine learning potentially presents a way to develop models reliably predicting individual outcomes for multifactorial and heterogeneous illnesses such as schizophrenia^17–21^. In clinical research, machine learning, or pattern recognition, refers to an algorithm that is able to learn from a large multivariate dataset to make an adequate prediction for a patient, for example concerning the future clinical outcome. Modern prospective multicenter studies facilitate the development of prediction models based on machine learning. They provide well-established outcome measures and large numbers of potential predictors (i.e. “features”), in study samples large enough to cover the heterogeneity of the target population^19^. A landmark study by Koutsouleris et al. recently demonstrated the potential of machine learning for individual outcome prediction in psychosis^18^. Pre-treatment data from a multicenter clinical trial were used to predict global outcomes after 4 and 52 weeks of treatment in first-episode psychosis. Predictive accuracy was found significantly above chance, at 73.8–75.0%. With an average drop of 2.8%, accuracy was retained when the models were tested on geographic sites left out of the model training procedure, suggestive of its validity in other samples. Unemployment, lower education, functional deficits, and unmet psychosocial needs were found most valuable in predicting 4- and 52-week outcomes.

Here, we extend the use of data-driven model development based on patient reportable data, to long-term (3 and 6 years) symptomatic and global outcomes of patients with schizophrenia-spectrum disorders. To this end, we include a heterogeneous population of schizophrenia-spectrum patients, with variable illness duration and baseline clinical status from the Genetic Risk and Outcome in Psychosis (GROUP) cohort study^22^. We explore the use of a wide range of baseline markers of genetic and environmental risk and measures of past and baseline clinical state, to predict 3- and 6-year symptomatic and global outcomes. We use data-driven selection to arrive at a model containing predictors from a limited number of measures, aiming at clinical applicability. We assess its generalizability by using leave-one-site-out (LOSO) cross-validation, testing our models on geographic study-sites left out of model development. Additionally, we investigate the use of the features that have been found to predict 4- and 52-week outcomes of first-episode psychosis^18^, for 3- and 6-year outcomes in the GROUP sample.

## Results

### Sample characteristics

We included 523 patients with a schizophrenia spectrum disorder who had outcome assessments three (*T*_3_) and six (*T*_6_) years after baseline. Demographic and clinical baseline characteristics of the study sample and comparisons to patients excluded because of missing follow-up assessments are listed in Table 1. Patients with unfavorable baseline characteristics were more likely to be lost to follow-up. At baseline, *T*_3_ and *T*_6_, 49%, 37%, and 41% of patients were in symptomatic remission (according to the consensus definition by Andreasen et al. (2005)) respectively; 31%, 44%, and 36% had good global functioning status (Global Assessment of Functioning (GAF) scale ≥ 65) at respective measurements. For symptomatic outcome, 65% and 64% of patients were stable at *T*_3_ and *T*_6_ relative to baseline, and 68% and 68% for global outcome (Supplementary Fig. 2).

**Table 1:** Baseline demographic and clinical characteristics of patients who completed baseline and follow-ups and of those who were not included in the study.

	Included patients (*n* = 523)	Excluded patients (*n* = 577)	*P* value
Age in years, mean (SD)	27.6 (7.4)	26.6 (7.0)	0.018
No. (%) male sex	402 (76.9)	426 (77.6)	0.775
No. (%) white ethnicity	449 (85.9)	363 (72.6)	<0.001
WAIS IQ, mean (SD)	97.4 (16.1)	92.1 (15.6)	<0.001
Education patient, mean (SD)	4.3 (2.0)	3.8 (2.1)	<0.001
Education father; SES, mean (SD)	5.1 (2.5)	4.7 (2.6)	0.014
Education mother; SES, mean (SD)	4.4 (2.4)	4.1 (2.5)	0.054
No. (%) employed/student	241 (46.1)	184 (41.2)	0.124
Illness duration in years, mean (SD)	4.6 (4.2)	3.9 (3.4)	0.002
No. (%) recent onset of psychosis in the past year	101 (19.3)	168 (30.7)	<0.001
No. (%) DSM-IV schizophrenia diagnosis, 295.1,2,3	342 (65.4)	341 (62.5)	0.317
No. (%) antipsychotic drug use present state	479 (91.6)	434 (99.3)	<0.001
No. (%) clozapine use present state	64 (12.2)	81 (14.8)	0.228
No. (%) cannabis abuse/dependency present state	160 (30.6)	179 (32.6)	0.479
No. (%) other illicit drug use in the past	324 (62.9)	365 (69.5)	0.024
PANSS positive symptoms, mean (SD)	12.2 (5.1)	13.3 (5.5)	0.001
PANSS negative symptoms, mean (SD)	13.3 (5.5)	14.7 (6.3)	<0.001
PANSS general symptoms, mean (SD)	27.0 (7.8)	29.0 (8.8)	<0.001
PANSS total, mean (SD)	52.4 (15.7)	56.9 (17.5)	<0.001
Global assessment of functioning; symptoms, mean (SD)	57.9 (16.0)	53.5 (15.3)	<0.001
Global assessment of functioning; degree of disabilities, mean (SD)	57.0 (15.6)	51.3 (15.8)	<0.001
No. (%) GAF score ≥65	173 (33.1)	94 (21.2)	<0.001
CAPE frequency symptoms, mean (SD)	0.9 (0.5)	0.9 (0.5)	0.267
CANSAS number of needs, mean (SD)	6.7 (3.8)	7.8 (3.9)	<0.001

*WAIS IQ* Wechsler Adult Intelligence Scale Intelligence Quotient, *SES* socioeconomic status, *DSM-IV* Diagnostic and Statistical Manual of Mental Disorders 4th edition, *PANSS* Positive and Negative Syndrome Scale, *GAF* global assessment of functioning, *CAPE* community assessment of psychic experiences, *CANSAS* Camberwell Assessment scale of Need Short Appraisal Schedule.

### Selection of modalities based on unimodal models

We included demographic information, illness-related variables, Positive and Negative Syndrome Scale (PANSS; present state clinician-rated symptomatology), and either Camberwell Assessment scale of Need Short Appraisal Schedule (CANSAS; clinician-rated and self-reported need of care) or Community Assessment of Psychic Experiences (CAPE; self-reported lifetime psychotic experiences) data for multimodal modeling. Notably, this set is especially rich on indicators of clinical course until inclusion in GROUP (i.e. includes GAF, features from PANSS, and CAPE where applicable). The choice of these modalities was based on unimodal modeling performance for the following modalities: (1) demographic variables; (2) illness-related variables; (3) PANSS; (4) substance use characteristics; (5) neurocognitive task scores; (6) social cognitive task scores; (7) Premorbid Adjustment Scale items; (8) CANSAS; (9) CAPE; (10) extrapyramidal symptoms; (11) genetic features, and familial loading of psychotic disorder, bipolar disorder, and drug abuse; (12) environmental variables of urbanicity and living situation (see the “Methods” section; Supplementary Tables 2 and 4). As mentioned earlier, we additionally trained models using a prespecified set of features that had performed best in predicting 4- and 52-week outcome of first-episode psychosis in the EUFEST study (22 and 24 features, respectively, see part C in Table 2, see the “Methods” section; Supplementary Table 1)^18^. A summary of the number of features and sample size for unimodal and multimodal models per outcome, and good versus poor outcome distributions is provided Supplementary (Supplementary Tables 2 and 3).

**Table 2:** Internal validation with nested cross-validation and leave-one-site-out (LOSO) nested cross-validation predicting symptomatic and global outcome at *T*_3_ and *T*_6_ in multimodal models.

Predictor/Model (outcome)	Internal BAC	Internal Sens/Spec	Internal PPV/NPV	Internal AUC	LOSO BAC	LOSO Sens/spec	LOSO PPV/NPV	LOSO AUC
PANSS, ill, demo, CANSAS (symptomatic outcome *T*_3_)	62.2 (1.7)	77.9/42.6	68.9/54.1	0.60	61.3	59.7/62.9	73.1/47.7	0.61
PANSS, ill, demo, CAPE (symptomatic outcome *T*_3_)	64.4 (1.9)	76.0/50.0	72.2/54.8	0.63	63.8	62.9/64.7	75.8/48.5	0.65
PANSS, ill, demo, CANSAS (symptomatic outcome *T*_6_)	64.7 (2.0)	78.7/46.5	69.0/59.0	0.63	62.5	68.5/56.5	74.3/49.3	0.62
PANSS, ill, demo, CAPE (symptomatic outcome *T*_6_)	62.3 (1.8)	75.4/46.5	66.2/57.6	0.61	59.9	64.2/55.6	69.3/49.1	0.62
PANSS, ill, demo, CANSAS (global outcome *T*_3_)	63.5 (1.9)	66.3/59.7	65.6/60.5	0.63	63.5	66.1/60.8	68.5/59.6	0.62
PANSS, ill, demo, CAPE (global outcome *T*_3_)	67.6 (1.3)	74.9/58.4	70.1/64.2	0.67	64.8	65.8/63.8	72.7/56.5	0.58
PANSS, ill, demo, CANSAS (global outcome *T*_6_)	67.6 (2.2)	81.8/47.7	71.8/61.6	0.65	64.0	71.8/56.1	73.2/55.3	0.64
PANSS, ill, demo, CAPE (global outcome *T*_6_)	67.3 (1.7)	84.3/43.3	73.4/59.8	0.64	61.2	65.9/56.5	76.8/45.5	0.64
	**External BAC**	**External Sens/Spec**	**External PPV/NPV**	**External AUC**				
EUFEST 4 weeks (symptomatic outcome *T*_3_)	62.7	61.3/64.0	69.0/45.1	0.62	–	–	–	–
EUFEST 52 weeks (symptomatic outcome *T*_3_)	59.0	60.9/57.1	70.5/47.4	0.60	–	–	–	–
EUFEST 4 weeks (symptomatic outcome remission *T*_6_)	62.4	58.1/66.7	72.0/50.9	0.62	–	–	–	–
EUFEST 52 weeks (symptomatic outcome *T*_6_)	61.0	60.2/61.8	69.8/50.0	0.60	–	–	–	–
EUFEST 4 weeks (global outcome *T*_3_)	60.4	61.7/59.1	64.5/57.9	0.61	–	–	–	–
EUFEST 52 weeks (global outcome *T*_3_)	56.5	58.5/54.6	60.3/53.4	0.57	–	–	–	–
EUFEST 4 weeks (global outcome *T*_6_)	62.0	61.4/62.7	73.2/50.3	0.62	–	–	–	–
EUFEST 52 weeks (global outcome *T*_6_)	66.4	70.0/62.8	76.1/57.1	0.67	–	–	–	–

*BAC (mean (SD))* is balanced accuracy (i.e. the average of sensitivity and specificity), *sens* is sensitivity, *spec* is specificity, *PPV* is positive predictive value, *NPV* is negative predictive value, *AUC* is area under the curve, *T*_3_ is follow-up at 3-years interval after the baseline, *T*_6_ is follow-up at 6-years interval after the baseline, *PANSS* Positive and Negative Syndrome Scale, *ill* illness related, *demo* demographic, *CANSAS* Camberwell assessment of need short appraisal, *CAPE* Community Assessment of Psychic Experiences, *EUFEST* *4 weeks* is set of 10% best performing features of 4-week outcome prediction of the European First Episode Schizophrenia Trial, *EUFEST* *52 weeks* is set of 10% best performing features of 52-week outcome prediction of the European First Episode Schizophrenia Trial.

**Table 3:** Important baseline features by model.

Baseline (*T*_0_) feature	Symptom outcome *T*_3_^1^	Symptom outcome *T*_3_^2^	Symptom outcome *T*_6_^1^	Symptom outcome *T*_6_^2^	Global outcome *T*_3_^1^	Global outcome *T*_3_^2^	Global outcome *T*_6_^1^	Global outcome *T*_6_^2^
ILL GAF disabilities	−	−	o	−	−	−	−	−
ILL GAF symptoms	o	−	−	−	−	−	−	−
PANSS poor judgment and Insight	+	+	+	+	o	+	+	o
PANSS hallucinatory behavior	+	+	×	+	+	o	+	+
PANSS flat affect	o	o	+	+	+	+	×	+
PANSS motor retardation	o	o	o	+	+	+	o	+
PANSS unusual thought content	o	o	o	+	+	+	o	+
ILL (health related) quality of Life	−	o	o	o	o	−	−	−
ILL status antipsychotics	o	o	+	+	o	o	+	o
ILL diagnosis schizophrenia/psychosis related disorders	−	−	−	o	o	o	o	o
PANSS passive/apathetic Social withdrawal	o	o	o	o	+	+	o	+
DEMO age	+	+	o	o	o	o	o	o
CANSAS number of met need	−		o		−		−	
CANSAS housing need	o		o		+		+	
CANSAS food need	o		−		+		o	
CANSAS number of no need	o		o		−		o	
CANSAS psychotic disorder unmet need	o		+		o		o	
CAPE feeling guilty		−		o		o		o
CAPE feeling tense		o		o		+		o
CAPE suicidal		+		o		o		o
CAPE lack of activity		o		+		o		o
CAPE hallucinations		o		+		o		o
CAPE telepathy		o		o		o		+
PANSS delusions	o	o	+	+	o	o	o	o
PANSS Suspiciousness/persecution	+	+	o	o	o	o	o	o
PANSS grandiosity	o	o	o	o	o	+	o	+
PANSS depression	o	+	o	o	o	o	o	+
PANSS lack of spontaneity	o	+	o	+	o	o	o	o
PANSS stereotyped thinking	o	o	o	o	+	+	o	o
PANSS difficulty abstract thinking	o	o	o	+	o	+	o	o
PANSS emotional withdrawal	o	o	o	+	o	+	o	o
PANSS tension	o	o	o	o	o	o	+	+

Important baseline features: selected in at least one-fourth of the models’ top 10% most frequently selected features. ^1^Models contained PANSS, demographic, illness, and CANSAS features; ^2^Models contained PANSS, demographic, illness, and CAPE features. +: positive weight; −: negative weight; o: not selected in the top 10% most frequently selected features; empty cell: not included in the model. Note that low weights (or beta’s): ≤0.10 were not considered in this Table (see Supplementary Tables 5–12 for specific weights). Weights (−/+) are relative to poor outcomes (i.e. “positive” outcome).

*Symptom* symptomatic, *T*_3_ follow-up at 3-year interval after the baseline, *T*_6_ is follow-up at 6-year interval after the baseline, *PANSS* Positive and Negative Syndrome Scale, *CANSAS* Camberwell assessment of need short appraisal, *CAPE* Community Assessment of Psychic Experiences.

### Performance of prediction models: Internal validation

Using a repeated nested cross-validation design (see the “Methods” section, Creation of models (A)) including recursive feature elimination, support vector machine models were trained to predict individual patient’s outcomes based on their baseline data from four modalities. The outcome could be predicted with similar cross-validated balanced accuracies (BACs), regardless of the period, outcome, and the fourth modality included (i.e. CANSAS or CAPE), ranging from 62.2% to 67.6% (Table 2). Model performance was well above benchmark models containing a single feature (GAF) and using threshold values for good/poor global functioning above and below GAF 65 did not result in higher model performance (Supplementary Note 4, Supplementary Table 15).

**Fig. 1:**
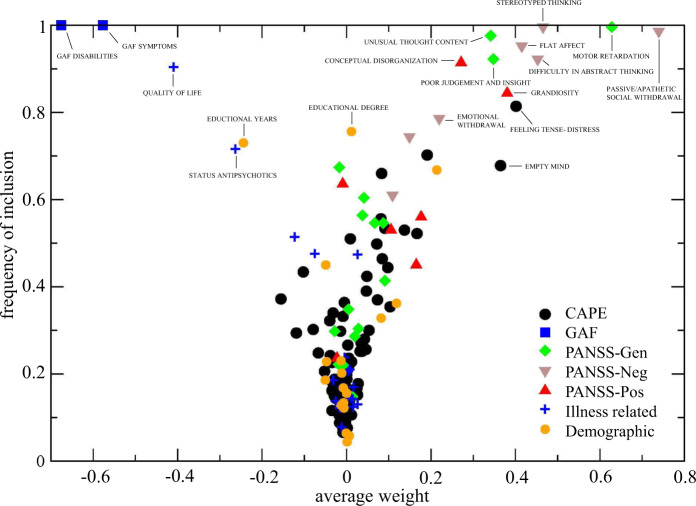
Frequency of inclusion and weight of features. Frequency of inclusion of a feature against its (average) weight in the model; shown for prediction of global assessment of functioning (GAF) outcome at *T*_3_, containing Positive and Negative Syndrome Scale—general, negative, and positive subscale (PANSS—Gen, Neg, and Pos), Demographic, Illness-related and lifetime psychotic experiences (CAPE) related features. A positive weight reflects that scoring higher on this feature contributes to being classified as ‘poor outcome’. For features with negative weights the opposite holds.

The 10% most influential features for symptomatic as well as global outcome based on weight and frequency of selection included items from all four modalities in the model: PANSS items, illness-related, demographic features, and either CANSAS or CAPE items (Supplementary Tables 5–12). As illustrated in Fig. 1, generally, the more often a feature is selected, the higher its average weight is (see Supplementary Figs. 3 and 4 for overviews of selection frequency over weight for all models).

Worse GAF symptoms and GAF disabilities, worse scores on specific items in the positive and negative subdomains of the PANSS (i.e. judgment and insight, hallucinatory behavior, flat affect, unusual thought content, motor retardation), worse score on (health-related) quality of life and the use of antipsychotics were associated with multiple poor outcome endpoints. This was supplemented by a lower number of no needs and met needs, together with housing needs and unmet psychotic disorder needs in models including CANSAS items. In models including the CAPE, items of importance from the CAPE mostly included those from the depression subscale (i.e. guilty and tense feelings, suicidal thoughts, and lack of activity) (Table 3).

The following features were predictive of at least one-fourth of poor outcome endpoints, albeit selected in fewer models than those summed in the previous paragraph: higher age, schizophrenia diagnosis, and a higher level of various present state symptoms in PANSS subdomains of positive, negative, disorganization symptoms and emotional distress (i.e. delusions, suspiciousness/persecution, grandiosity, stereotyped thinking, lack of spontaneity, difficulty in abstract thinking, emotional withdrawal, depressive symptoms, and tension) (Table 3).

Comparison of frequently misclassified patients to those mostly correctly predicted showed the following: patients with a good outcome, who were incorrectly classified as having poor outcome (21–37% over the models) showed less favorable baseline characteristics (e.g. higher PANSS, lower GAF scores, more chronicity, lower parent education, and more schizophrenia diagnoses) when compared to the correctly classified group of patients. Contrary, patients with poor outcome who were mostly incorrectly classified as having good outcome (3–14%) showed favorable baseline characteristics, such as lower PANSS and higher GAF scores, when compared to the most correctly classified group of patients (see Supplementary Table 13 for a detailed overview of significant comparisons).

### Generalization of the prediction models: LOSO validation

The generalizability of the models was evaluated by consecutively leaving out patients from one of the four geographic sites and testing the model trained on the three remaining sites in these patients (see the “Methods” section, Creation of models (B)). These LOSO validated models had slightly (−2.3% on average) lower accuracies than models trained on the full dataset (Table 2). The range of the average prediction accuracies for symptomatic outcome at *T*_3_ and *T*_6_ was 59.9–63.8% (Table 2); site-specific BACs ranged from 53.0% to 69.7% (Table 4). For global outcome the range was 61.2–64.8% (Table 2); BACs of the different sites ranged from 53.0% to 68.9% (Table 4). The difference between *T*_3_ and *T*_6_ prediction accuracy was small (mean BACs were 63.4% and 61.9%, respectively). There was, again, a small difference between CANSAS-based and CAPE-based models (mean BACs were 62.8% and 62.4%, respectively). LOSO models validated on the Utrecht site and single-site models trained on the Utrecht site tended to perform below average, in line with the differential patient profile (higher baseline symptom severity, lower GAF scores, and higher needs) found at this site relative to the other geographic sites and its smaller sample size (Supplementary Note 4.4; Supplementary Table 14).

**Table 4:** Leave-one-site-out cross-validation site performance by model.

	***N***	**BAC Symptomatic outcome** ***T***_3_^1^	***N***	**BAC Symptomatic outcome** ***T***_3_^2^	***N***	**BAC Symptomatic outcome** ***T***_6_^1^	***N***	**BAC Symptomatic outcome** ***T***_6_^2^
Amsterdam	81	60.8	104	69.5	81	69.3	104	60.1
Groningen	73	62.4	132	63.1	73	62.3	132	57.2
Maastricht	124	56.4	139	53.0	124	63.8	139	61.5
Utrecht	54	65.5	70	69.7	54	54.4	70	60.6
	***N***	**BAC Global outcome** ***T***_3_^1^	***N***	**BAC Global outcome** ***T***_3_^2^	***N***	**BAC Global outcome** ***T***_6_^1^	***N***	**BAC Global outcome** ***T***_6_^2^
Amsterdam	80	66.4	100	68.1	77	63.9	98	65.4
Groningen	65	61.5	118	64.4	58	62.5	107	64.2
Maastricht	81	68.0	93	68.9	124	66.3	139	62.1
Utrecht	48	58.0	66	57.9	54	63.1	70	53.0

Rows mention the geographic site left out of model training. Columns mention models organized per timepoint and included modalities. ^1^Models contained PANSS, demographic, illness-related, and CANSAS features; ^2^Models contained PANSS, demographic, illness-related, and CAPE features.

*BAC* balanced accuracy, *T*_3_ follow-up at 3-year interval after the baseline, *T*_6_ is follow-up at 6-year interval after the baseline, *PANSS* Positive, and Negative Syndrome Scale, *CANSAS* Camberwell assessment of need short appraisal, *CAPE* community assessment of psychic experiences.

### External validation of EUFEST predictors

Predicting long-term outcome based on the top 10% most predictive features for the short-term outcome (EUFEST study (see the “Methods” section, Creation of models (C))), resulted in accuracies of 59.0–62.7% for symptomatic outcome. For global outcomes, we obtained accuracies of 56.5–66.4% (Table 2).

## Discussion

Using a rigorous machine learning approach, we developed individualized models to predict 3- and 6-year symptomatic and global outcomes of patients with schizophrenia-spectrum disorders based on patient-reportable data. The multicenter sample included 523 schizophrenia-spectrum patients with variable illness duration, mainly with established illness. Notably, baseline clinical status was variable, and outcome status remained poor at follow-up in a large share of patients. The data-driven nature of this study allowed us to explore the predictive value of a wide range of measures for the long-term outcome of psychosis. In keeping with clinical applicability, our aim was to arrive at lean models. We report nested-cross-validated balanced accuracies ranging from 62.2% to 67.6%. Suggestive of generalization of model performance to out-of-study samples, leave-site-out cross-validation showed minor drops in accuracy, with balanced accuracies ranging from 59.9% to 64.8%. Models trained in our sample for long-term outcome prediction, utilizing short-term outcome predictors for first-episode psychosis^18^, yielded comparable balanced accuracies up to 66.4%.

To the best of our knowledge, no prognostic models for the long-term global and symptomatic outcomes of psychosis are presently available^23^. Our results indicate that while state-of-the-art methods may result in robust (generalizable) performance estimates, predictions are modestly accurate, similar to recent experimental prognostic models for depression based on machine learning predicting long-term clinical outcomes based on patient reportable data^17,24^. The models did not reach the LOSO cross-validated accuracy of 71% in the study on the one-year outcome of first-episode psychosis^18^, presumably due to the uncertainty introduced by time, care-as-usual setting, and the heterogeneity of baseline clinical status and illness duration within our target population.

Through a modality-wise learning strategy, a combination of baseline sociodemographic features and clinician-rated symptoms, complemented by self-rated lifetime psychotic experiences (CAPE items) or psychosocial needs (CANSAS items) was selected in the models. Interestingly, in unimodal models, these state-based and context-based modalities outperformed trait-based modalities, including genetic and cognitive task scores. This finding may be partly explained by the relatively large share of patients with a stable clinical state at inclusion and follow-up. We further argue that the performance of trait-based measures may improve if the interaction between genetics and environmental exposures in psychosis outcomes is taken into account^25^.

Features offering a clinician’s integration of the clinical picture and those with a broad underlying construct (e.g. GAF; insight; schizophrenia diagnosis; quality of life; summed no need/met need items; depression) show to be the most important predictors. These resemble features found to be predictive of one-year outcome in first-episode psychosis: psychosocial needs, global functioning deficits, and affective symptoms (specific quality of life, CAPE, and PANSS items)^18^.

In comparison to the aforementioned study, and our work, we also note differences, suggesting differential ways to short-term and long-term clinical management of psychosis. We found higher, and not lower symptom severity to predict poor long-term outcome^18^. In particular, lack of insight appears predictive of poor long-term outcomes across all the models. This may be mediated through poor adherence and eventual service disengagement^26^. Furthermore, we note that the most important social need in our models (i.e. housing) is different from those (company, daytime activities) predicting short-term outcomes. This could be explained by the lower level of social functioning found in our study cohort, compared to first-episode patients^27^, suggesting that in a model suited for a functionally heterogeneous population, the entire range of social needs within the CANSAS instrument may have its relevance. In interpreting the influence of features on the predictions, it should be noted that some, frequently selected, features show large variation in weight. This variation could have its origin in the heterogeneity of the disorder and should be the subject of future research.

Within our models, misclassification especially occurs in patients with unfavorable clinical baseline status combined with good outcomes. This may reflect variation in baseline clinical context and acute state at the time of inclusion (i.e. admission due to relapse vs. outpatient treatment) of these patients and/or availability of therapeutic or supportive resources. We note that the higher baseline symptom severity, lower GAF, and higher psychosocial needs found in one geographic site that underperformed in the LOSO validation relative to the others, may support his possibility. To enhance model performance, these contextual factors may be taken into account in future models.

Our models on long-term outcomes of psychosis perform with reasonable accuracies, but at present are not suitable as a stand-alone tool to stratify treatment. Regardless, the machine learning model trained here and a clinician would represent rather different takes on reality. The model sees the patient through the lens of a number of constructs, such as “insight”, whereas a clinician’s judgment is more globally constructed and starts from the moment the clinician meets his patient in the waiting room^28,29^. Models with modest accuracy may be of use, depending on the level of uncertainty parties involved in clinical decision-making are willing to accept from a model^30,31^, a level which to our knowledge is unknown for long-term outcomes of psychosis. In addition, the preferable way of interaction between model and clinician remains to be addressed^32^. We suggest clinicians may inform their decision making, both by the prediction itself and important features in it, for example, high core social needs, affective symptoms, or low quality of life. Apart from clinical practice, modestly accurate model predictions may serve intervention research by offering stratified randomization.

Future prediction tool development should be informed by end-user (i.e. patients and clinicians) needs concerning scope, predictive capacities, and potential clinical consequences. We need to learn how they weigh benefit and harm due to treatment choices against a given outcome probability^30^. Furthermore, the significance of any predicted outcome might differ per patient, per stage of illness, and per intervention^10^. Hence, presenting an array of outcome dimensions with accessible features might best fuel the clinician–patient dialog on intervention^33^. Moreover, clinical guidance on when and how to use prognostic tools might prove essential for future dissemination of prognostic models based on machine learning in psychiatry.

We note the following limitations. Although we present the largest machine learning study to date on outcome in psychosis, based on patient-reportable data, the sample size may not be sufficient to account for the substantial heterogeneity of the out-of-study population with a schizophrenia-spectrum disorder^34^. It should be noted that the drop in performance was small (on average 2.3%), when the models were applied to patients from geographic sites not part of the training sample, suggesting transportability to samples with a comparable profile. Although we implemented a comprehensive validation procedure, we cannot rule out some overfitting not accounted for^35^, including that resulting from information leakage because modality selection, imputation, and scaling were performed outside the nested cross-validation pipeline. Apart from this, our approach of taking the four best performing data modalities from unimodal modeling runs together does not necessarily yield the best performing combination in a multimodal model. Instead, models may benefit from a combination of modalities containing a wider range of information, as has been suggested by studies combining patient reportable and imaging data^36,37^. We suggest future research may address what is a clinically parsimonious set of modalities, that is, an optimum between accuracy and investment to obtain data. Furthermore, we believe that imputation and scaling outside the cross-validation setup has not led to over-optimistic estimates of generalizability, because of the very low number of imputations (<0.5%) and the fact that most of our features’ scales are fixed, thus independent of our dataset.

The GROUP study sample is known to represent a relatively well functioning subset of a population of schizophrenia-spectrum patients in need of specialist care. Generalization to other samples further might be hindered by the exclusion of the most severely affected patients, either due to study drop-out, exclusion of patients with extensive missing data, or incompetence or unwillingness to give study consent^38^. The nature of the sample included may also explain the association of antipsychotics use with worse outcomes, as antipsychotics use at baseline is likely to be confounded by history or expectation of more severe illness course. The observational sample obtained may however be more representative for clinical practice than those stemming from clinical trials. To improve model reliability, the use of multi-center samples dedicated to prognostic model development or models informed by national registry data, as has been done to predict transition to psychosis from high-risk mental states and suicidal behavior, may be needed^39–41^.

Regarding outcomes, prediction of longitudinal patterns, or adverse events, such as readmission to a psychiatric hospital may also add clinical relevance, especially for long-term outcome^42^. We used baseline data for outcome prediction, whereas in clinical practice, decisions are typically based on longitudinal, rather than single, examinations. Longitudinally informed models are expected to result in better prediction accuracies. Furthermore, we propose that contextual information, such as baseline clinical context (e.g. acute inpatient or outpatient status, treatment status), or supportive resource status (e.g. family support) may further enhance model performance. The addition of biomarker modalities, including imaging data and genetic data derived from genome-wide association studies, possibly in interaction with environmental exposures, holds the same promise^41^. However, all additions come at the expense of time investment, model interpretability, and the requirement of larger training datasets^20^.

In conclusion, we demonstrate the feasibility of a machine-learning approach to long-term outcome prediction in a heterogeneous target population of schizophrenia-spectrum patients, based on a lean set of patient reportable features, overlapping with those predictive of short-term outcome of first-episode psychosis. Future models may benefit from considering patient’s and clinician’s needs, the appropriate nature of the training sample (i.e. sample similarity to the population of interest as well as richness on (contextual) features), and implementation of advancements in machine learning methodology. Individual outcome prediction based on machine learning may inform the treatment stratification needed both from a patient and a public health perspective.

## Methods

### Participants and data selection

In the GROUP prospective longitudinal cohort study, in- and out-patients with a psychotic disorder presenting consecutively at selected representative mental health services in representative geographical areas in the Netherlands and Belgium from January 8, 2004 until February 6, 2008 were recruited. Inclusion criteria were: (1) psychotic disorder diagnosis according to the Diagnostic and Statistical Manual of Mental Disorders, Fourth Edition APA^43^; (2) age 16–50 years (extremes included); (3) Dutch language proficiency; (4) ability to provide informed consent. Extensive genetic, cognitive, environmental, and outcome data were collected at baseline (*T*_0_), and 3-year (*T*_3_) and 6-year (*T*_6_) follow-up. The full GROUP sample at baseline included 1119 patients with variable illness duration, including recent-onset psychosis^22^. Here, we used data of 523 participants for whom outcome assessments at both *T*_3_ and *T*_6_ were available, with a schizophrenia spectrum disorder (i.e. schizophrenia, schizophreniform disorder, schizoaffective disorder, delusional disorder, brief psychotic disorder, psychotic disorder: not otherwise specified), assessed with the Comprehensive Assessment of Symptoms and History or the Schedules for Clinical Assessment for Neuropsychiatry (see Supplementary Fig. 1 for a selection process flow-chart)^44,45^. We assessed selection bias by comparing our sample on demographic and clinical characteristics to GROUP patients not included in this study.

The study protocol was approved by the Medical Ethical Review Board of the University Medical Centre Utrecht and by local review boards of participating institutes. Participants provided written informed consent. Database release 5.0 was used in all analyses.

### Long-term outcomes and baseline predictors

We selected two long-term outcome measures in a classification approach to outcome prediction: symptomatic remission and global functioning, measured at both *T*_3_ and *T*_6_. Symptomatic outcome was selected as it traditionally is a mainstay of clinical care. We followed the consensus definition of symptomatic remission by Andreasen et al., operationalized as a mild score (3 or less, implying no functional disturbance related to symptoms) on selected items of the PANSS (i.e. delusions, conceptual disorganization, hallucinatory behavior, mannerism and posturing, blunted effect, social withdrawal, lack of spontaneity and unusual thoughts), maintained for at least 6 months^46,47^. For global outcome we followed Koutsouleris et al. in operationalizing global outcome with a dichotomization of the Global Assessment of Functioning (GAF) scale, considering a score of <65 poor global outcomes and ≥65 good global outcomes^18,43^, as GAF scores between 61 and 70 have been proposed as a threshold between at-risk mental states and illness and widely used as markers of recovery as part of more complex criteria^48,49^. GAF was constructed as a mean composite score of the GAF symptoms and GAF disabilities subscales assessed in the GROUP project, and normally distributed in our sample. To investigate the possibility that a threshold other than GAF 65 would better represent a border between “good” and “poor” outcomes, we tested other cut values in the GAF 50–68 range post-hoc (Supplementary Note 4.2).

All clinical variables assessed at baseline within the GROUP project which permitted a sample size >250 patients were considered for inclusion as a predictor, barring the models which contained a prespecified set of features based on the best-performing features in the EUFEST study by Koutsouleris et al. (see Supplementary Table 1)^18^. We clustered available candidate baseline predictors in modalities according to information type: (1) demographic variables, including age, sex, education, socioeconomic status, living situation, and employment; (2) illness-related variables, of diagnosis, comorbidities, illness course duration of untreated psychosis, quality of life and medication use; (3) clinician-rated, present state symptoms as measured by the PANSS^47^; (4) substance use characteristics (i.e. illicit drug use, alcohol use and smoking) indicated by urine analysis and the Composite International Diagnostic Interview^50^; (5) neurocognitive task scores of IQ, memory, processing speed/attention and executive functioning, assessed with the Wechsler Adult Intelligence Scale-Third Edition short form, Word Learning Task, Continuous Performance Task-HQ and Response Set-shifting Task respectively; (6) social cognitive task scores of theory of mind, affect recognition and facial recognition, assessed with Hinting Task, Degraded Affect Recognition Task and Benton Facial Recognition Test respectively. For psychometric instrument references for cognitive testing, see Supplementary Note 1.5; (7) Premorbid Adjustment Scale items^51^, comprising social and cognitive functioning in childhood and adolescence; (8) need of care items, measured with the CANSAS^52,53^; (9) self-rated lifetime psychotic experiences, consisting of Community Assessment of Psychic Experiences questionnaire (CAPE) items^54^; (10) extrapyramidal symptoms, comprising akathisia, dyskinesia, and Parkinsonian symptoms; (11) genetic features (i.e. polygenic risk score for schizophrenia^55^, and familial loading of psychotic disorder, bipolar disorder and drug abuse, measures that comprise the absence or presence of affected relatives of the patient^56^; (12) environmental variables of urbanicity and living situation. For global content of, and features within the modalities, see Supplementary Note 1 and Supplementary Table 1). Within each modality, missing data for every feature and subject with <20% missing values was imputed and scaled; features and subjects with ≥20% missing values were excluded (also see Supplementary Note 2).

### Creation of individual prediction models: machine learning strategy

**Fig. 2:**
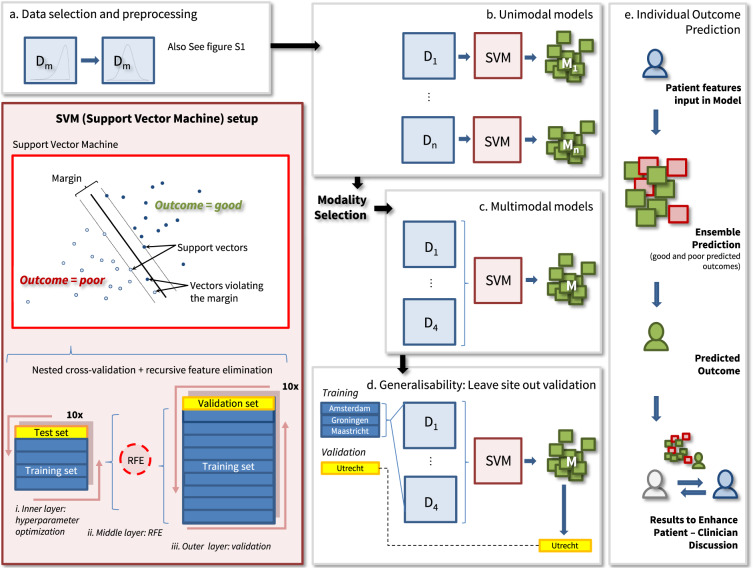
Machine learning training and validation design. Machine learning pipeline. D = modality; M = Model; training sets in dark blue, test/validation sets in yellow. **a** Data selection (see Supplementary Fig. 1 for details) and preprocessing including scaling and imputation. **b** Unimodal models, to identify the most informative modalities. **c** Multimodal models consisting of 2–4 modalities, including recursive feature elimination (RFE). **d** External validation of multimodal models using leave-one-site-out (LOSO) validation, where one of the four geographic sites is held out of model training and used for external validation; SVM (support vector machine) setup: RFE is part of the SVM pipeline; (i) In the inner layer, a CV loop is used to find the optimal value for the cost hyperparameter C from 38 points equidistant in ^2^log, starting at 0.0001 and ending at 37.07. C sets a penalty for violating the margin of the hyperplane; (ii) the middle layer employs a CV loop for RFE, a feature selection algorithm. It starts by including all available features in the model and iteratively eliminates the least informative features from it until the stopping criterion is met. The smallest set of features with performance within 10% of the best-performing set is selected; (iii) in the outer layer, a CV loop is used to define feature weights in the training set (9/10th of the data) and test the accuracy of the model in the validation set (1/10th of the data). Repetition of this procedure yields 10 models, which are repeated 50 times to reduce dependency on the choice of train-test partitions. The final prediction for a patient is an ensemble constituting an average of 50 repetitions.

We trained a linear support vector machine (SVM)^57^, to find the optimal separating hyperplane dividing patients into the two outcome classes (Fig. 2). For a given training dataset, each patient is represented by a labeled datapoint in an *m*-dimensional feature space. The position of the data point is determined by the score on the *m* baseline predictors (input features) and its binary label is the outcome (−1: good outcome; +1: poor outcome). SVM returns feature weights, reflecting the relative influence of predictors on outcome prediction. We used weighting by outcome class to account for unbalance between outcome group sizes and blind the algorithm to base rate distribution, to avoid model bias towards the largest outcome group. Internal validation was performed with three-layer, 10-fold nested cross-validation, where the inner cross-validation layer optimized the cost parameter, representing a penalty imposed on cases violating the margin of the decision boundary of the model. The middle layer selected the smallest predictor set with performance within 10% of the best performing set by recursive feature elimination (RFE). The outer layer provided performance estimates, reflecting the accuracy of the ensemble of *k* models taken together. This validation procedure was repeated 50 times to reduce dependency on the choice of train-test partitions. We employed the e1071 library (version 1.6.8) for SVM in R (version 3.4.0); and the caret package (version 6.0.76) for RFE^58,59^.

See Supplementary Note 3 for an elaborate description of the machine learning pipeline, and Supplementary Note 4.3 for a comparison to an alternative nonlinear learning design (random forests classifier + IsoMap dimensionality reduction, implemented in Neuropredict^60^) which yielded comparable performance in the study sample (Supplementary Table 16).

### Creation of individual prediction models: training and validation design

We employed a data-driven, modality wise learning strategy with the aim of automatically identifying a concise set of features from a limited number of clinical instruments. We entered the best performing modalities from preliminary uni-modal modeling runs (Fig. 2b; Supplementary Table 2) together into the SVM to train a multi-modal prediction model (Fig. 2c).

To align with best practice in prognostic model development^19^, our study included three components: model development, model validation, and comparison to existing models. (A) Data-driven model development including internal validation using repeated 10-fold nested cross-validation (Fig. 2a–c). Single-feature models containing baseline GAF only as a predictor were additionally trained to benchmark model performance (Supplementary Note 4.1). (B) A test of generalization to out of study samples with leave-one-site-out (LOSO) validation (Fig. 2d). Each of the four geographical sites (Amsterdam, Utrecht, Groningen, and Maastricht) of the GROUP study was held out once, and the prediction model was trained on patients from the remaining three sites. This model was then tested on the hold-out site, to yield prediction accuracy in a site geographically distinct from sites the model was trained on. To estimate predictive power in unseen data, the average prediction accuracy from four LOSO-runs was calculated. We assessed differences between geographic sites on the measures included in the models and ran single-site models post-hoc, to offer possible explanations to performance differences between LOSO-runs (Supplementary Note 4.4). (C) Applicability of 4-week and 52-week outcome predictors in first-episode psychosis for 3- and 6-year outcome prediction in a heterogeneous sample. We selected GROUP predictors matching the top 10% 4- and 52-week global outcome predictors from the European First Episode Schizophrenia Trial (EUFEST; Supplementary Table 1)^18^, and trained the SVM testing their capability of predicting long-term outcomes within the GROUP sample.

We assessed model performance by calculating sensitivity, specificity, balanced accuracy (BAC: the average of sensitivity and specificity), positive predictive value, and negative predictive value. To give an overview of important features to predict long-term outcomes, we listed features with the highest selection chance per model (top 10%), selected in >1 model. Since the entire cross-validated RFE procedure was repeated 50 times we were able to calculate the percentage of misclassified and correctly classified patients within these 50 repeats. To explore ways to enhance future model performance, we compared the profile of ≥90% misclassified patients with that of ≥90% correctly classified patients on sociodemographic and clinical characteristics, separately for the poor and the good outcome groups. We made use of the Transparent Reporting of a multivariable prediction model for Individual Prognosis Or Diagnosis (TRIPOD) statement^61^.

### Reporting summary

Further information on research design is available in the Nature Research Reporting Summary linked to this article.

## Supplementary information

Supplementary Information

Reporting Summary

## Data Availability

The data that support the findings of this study are available on request from the corresponding author. The data are not publicly available due to them containing information that could compromise research participant privacy or consent.

## Code availability

The code used for analyzing the data is publicly available at: https://github.com/patterns-in-psychiatry/GROUP_outcome.
