# Supplementary material for: Individualized prediction of three- and six-year outcomes of psychosis in a longitudinal multicenter study: a machine learning approach
Source: NPJ Schizophr. 2021 Jul 2;7:34. doi: 10.1038/s41537-021-00162-3 (PMC8253813; doi:10.1038/s41537-021-00162-3)
Supplement: Supplementary file 1 — Supplementary Information [file 41537_2021_162_MOESM1_ESM.pdf]

Supplementary Information for 'Individualized prediction of three- and six-year outcomes of psychosis in a longitudinal multicenter study: a machine learning approach'.

Jessica de Nijs[§1], Thijs J. Burger[§2,3], Ronald J. Janssen[1], Seyed Mostafa Kia[1], Daniël P. J. van Opstal[1], Mariken B. de Koning[2,3], Lieuwe de Haan[2,3], GROUP investigators*, Wiepke Cahn[§1,4], Hugo G. Schnack[§1ǂ]

[§]equal contribution

*A list of authors and their affiliations appears in the main text of the paper

[ǂ]corresponding author

Affiliations

[1]University Medical Center Utrecht, Department of Psychiatry, UMC Utrecht Brain Center, Utrecht University, Utrecht, The Netherlands; [2]Arkin, Institute for Mental Health, Amsterdam, The Netherlands; [3]Amsterdam UMC, University of Amsterdam, Department of Psychiatry, Amsterdam, The Netherlands; [4]Altrecht, General Mental Health Care, Utrecht, The Netherlands

Supplementary Note 1. Description of predictors

Below, we describe the global content of different modalities of predictors; i.e. sets of variables assessed at baseline, which will be used to predict outcome at T_3 and T_6. See supplementary Table S1 for a specification of predictors per modality.

1.1. Socio-demographic variables

Baseline demographic characteristics of the patients, i.e. self-reported sex, age, ethnicity, (parental) education, living/family situation, employment and number of lifetime moves were assessed. Number of staying backs and whether a patient had received special education were recorded. In the Dutch educational system special education constitutes schools for disabled children, children with behavioral and / or psychiatric disorders and for children with cognitive

problems. Educational level and degree ranged from 0 to 8 (0: no education, 1: primary school, 2, 3: secondary school, 4: high school, 5, 6 vocational education, 8: university degree). Educational level and degree of both mother and father of the patient constituted the parental SES. Scoring regarding ethnicity was dichotomized: score could either be white or non-white/mixed. If the country of origin of three or more grandparents of the subject was similar, the subject's ethnicity was equal to this. In all other cases, the ethnicity was mixed. Lifetime postal codes were registered, thus the number of lifetime moves could also be extracted from the database. Living situation/household was either scored 'independent living' or 'dependent living'. Independent living included subjects with a single-person household, or those living with their partner and/or own family. Dependent living was defined as sheltered living, living with parents, or 'other' (i.e. hospital admission, homelessness, living with sibling(s)). Living with parents was considered as deviant from the norm as subjects were on average 27.6 (±7.4 S.D.) years old. Whether the patient lost a parent/parents and whether they have children was also registered.
Patients were asked whether they had any occupation. Employment was defined as having a paid job. Volunteer work, as a consequence, did not constitute employment in this study. Besides employment, occupation also included whether the participant was currently a fulltime student.

1.2. Illness related variables

Baseline illness related characteristics of the patients were registered, and included information on course of illness, duration of untreated psychosis, diagnostics, quality of life, comorbidity of lifetime depression and suicide attempt, antipsychotic drug use, degree of functioning and disabilities. Diagnostic subtypes (disorganized, catatonic, paranoid, residual and undifferentiated types of schizophrenia, schizophreniform disorder, schizoaffective disorder, delusional disorder, brief psychotic disorder and psychosis NOS), as rated with the Comprehensive Assessment of Symptoms and History (CASH) or the Schedules for Clinical Assessment for Neuropsychiatry version 2.1 (SCAN),[1,2] were converted into a binary variable: schizophrenia or other than schizophrenia within the schizophrenia spectrum. In a composite questionnaire the following illness related information was acquired: age of psychosis onset (AOP; which was categorized in: early AOP 0-19, normal AOP 20-30, late AOP >30), duration of illness, whether the illness onset was recent (i.e. in the past year and in the past two years), duration of untreated psychosis (i.e. age when first treated with antipsychotic drugs and age of

first contact with a mental care institute subtracted by the AOP) and course of illness (having had one episode, episodic (i.e. having had multiple episodes), chronic course and cases of recent onset in the past year and past two years). Global level of functioning was assessed with a mean composite score of the symptoms and disabilities subscales of Global Assessment of Functioning scale.[3] Also information on whether or not patients currently used clozapine was assessed. Quality of life and quality of health were assessed on a five-point scale with the World Health Organization Quality of Life- short version (WHOQOL-BREF).[4]

1.3. PANSS

In the GROUP project, current severity of symptoms was measured with the PANSS, which consists of 30 items.[5] Each item is scored on a seven-point scale ranging from 1 (absent) to 7 (extreme), with item rating incorporating the behavioral effect of symptoms severity and frequency. The PANSS consists of three subscales, each measuring positive, negative or general symptoms.

1.4. Substance use

Current and lifetime use of tobacco, alcohol and illicit drug was assessed with the Composite International Diagnostic Interview (CIDI; sections B, J and L respectively).[6] Urinalysis by an external laboratory revealed recent cannabis use. Cut-off level was 50 ng/ml. These substance use related features were dichotomized in ‘using’ or ‘not using’.

1.5. (Social) cognitive testing

Neurocognitive and social cognitive functioning was assessed with a test battery with a duration of 90-120 minutes. IQ was measured with the Wechsler Adult Intelligence Scale– Third Edition short form, using the subtasks: Arithmetic, Information, Digit–Symbol Coding and Block Design.[7] Processing speed and attention were assessed using reaction time and accuracy of the Continuous Performance Task–HQ respectively.[8,9] Executive functioning was measured using the Respons Shifting Task.[10] Short– and long–term verbal memory were assessed using the Word Learning Task with immediate recall and delayed recall as outcome measures.[11] Benton Facial Recognition was used to assess face recognition.[12] Degraded Facial Affect Recognition was used to assess emotion recognition,[13] and theory of mind was measured with the Hinting Task.[14]

1.6. Premorbid Adjustment Scale

Premorbid academic and social assessment was assessed with the Premorbid Adjustment Scale (PAS).[15] It was designed to retrospectively evaluate the degree of achievement of academic and social goals in three distinct age epochs: in childhood (0 to 12 years), early adolescence (12 to 16 years) and late adolescence (16 to 19 years). Academic adjustment consisted of school performance and school adaptation subscales and social adjustment consisted of social behavior, peer relations and social-sexual aspect subscales (i.e. social sexual aspects 12 to16 years, social sexual aspects 16 to19 years and from 16 to 19 year: independence, highest level of functioning, social personal adaptation, interest in life and energy level). Premorbid adjustment is scored on a 7-point scale ranging from 0 (best functioning) to 6 (worst functioning). Informants were either a parent, a family member of the patient or patients themselves.

1.7. CANSAS

The Camberwell Assessment scale of Need Short Appraisal Schedule (CANSAS) was used to assess need of care of a patient in the past three months and whether the need is met or unmet.[16,17] It comprises the question whether there is a need in 24 different clinical and social domains, and whether it is met or unmet according to the patient as well as the clinician. Assessment of need is scored on a 3-point scale (0: no problem, no need; 1: need, but resolved by care; 2: need but unmet by care) or rated as 9: unknown. If a need is established more information is gathered concerning the adequateness of the effect of the care received. If there is no consensus between the patient and the clinician whether there is a need the item is always scored either 1 or 2. If there is no consensus between the patient and the clinician whether the need is met or unmet, a 1 is scored only when the patient has unrealistic expectations about the care, and a 2 is scored when either the patient or the clinician considers the care met. Additionally, the amount of no need items, met need items and unmet items were calculated.

1.8. CAPE

The Community Assessment of Psychic Experiences (CAPE) assesses lifetime frequency and amount of distress of psychotic experiences.[18] It is a self-reported 42-item questionnaire. The CAPE is rated on a 4-point Likert scale, ranging from 0 (less frequent/distress) to 3 (most frequent/distress).

1.9. EPS

Extrapyramidal symptom assessment consisted of global clinical assessment of akathisia and diagnosis of dystonia. Dyskinesia was measured with items one to seven of the Abnormal Involuntary Movement rating Scale. With the unified Parkinson's disease rating scale Parkinsonian symptoms such as bradykinesia, rigidity and tremor were measured (for detailed explanation and references of the variables described in this paragraph see Korver *et al.*, 2012.[19]

1.10. Genetic contribution

For calculation of familial loading score of bipolar disorder, psychotic disorder and drug abuse, we used the method described by Derks *et al.*[20] In short, the absence or presence of affected relatives of the patient was assessed. Within the calculation of the familial loading score, the amount of affected relatives, the age and sex of the relatives and the degree of relatedness were taken into account. A polygenic risk score for schizophrenia was calculated following the methods described by McLaughlin *et al.*[21] We used a threshold of $p=.1$, including 121958 single nucleotide polymorphisms.

1.11. Environmental contribution

The environmental modality of predictors consists of the level of urbanicity (at birth and present state), the number of people living with the patient and whether or not there was any experience of maltreatment or assault before and after psychosis onset. The number of people living with the patient was assessed and constituted living with family members, with other patient in mental care institute, with housemates or alone. For the assessment urbanicity at birth and current urbanicity, participants were asked to report the postal codes were they had lived/live. These were then coupled to the national database of Statistics Netherlands to determine the level of urbanicity (i.e. population density in number of inhabitants/km^2).

Supplementary Note 2. Data selection and rescaling

Within each modality, we excluded predictor variables with ≥20% missing values and subjects if ≥20% of the data for that subject was missing. Remaining missing data were imputed, using an expectation maximization algorithm in the statistical software package IBM SPSS version 22.0.[22] In total, 539 (<0.5%) missing values out of 113,049 values were imputed. Since the data was missing at very low percentages and completely at random in each modality, (mean (S.D.)=1.0% (1.1%)) this is unlikely to be a problem.[23]

Most features had established minimum and maximum scores and were scaled as follows: $x = \frac{x - \text{scale minimum}}{\text{scale range}}$. Features with a continuous level of measurement were rescaled to normalize the range by subtracting the mean and dividing by two times the standard deviation. Age was scaled by dividing the age in years by 50.

While performing the imputation and scaling outside the cross-validation setup may potentially lead to data leakage, the very low number of imputations (<0.5%) and the fixed (thus independent of our dataset) scales of most of our features will result in hardly any overoptimistic estimates of the out-of-sample performance.

Supplementary Note 3. Machine learning pipeline

We trained a linear support vector machine (SVM) to find the most optimal separating hyperplane separating patients into the two outcome classes. For a given training dataset, each patient is represented by a labelled datapoint in an *m*-dimensional feature space. The position of the datapoint is determined by the score on the *m* baseline predictors (input features) and its binary label is the outcome (-1: good outcome, +1: poor outcome).

Internal validation was performed with three-layer, k-fold cross validation (CV). The inner CV layer optimized the cost parameter, from 38 points equidistant in 2log, starting at 0.0001 and ending at 37.07, representing a penalty imposed on cases violating the margin of the decision boundary of the model.

In the middle layer, in order to build sparse models, recursive feature elimination (RFE) was used. With RFE, as implemented in the caret package for R,[24,25] we recursively remove the least informative feature, as judged by the impact of its removal on the in-sample performance. In each of these steps, an SVM was trained using 10-fold CV. The elimination procedure took place inside a 10-fold CV loop. In each fold, the performance for each pruned model was estimated on the corresponding test set. We selected the smallest set of features with a performance that was within 10% of the best-performing set. This encourages sparse models, which tend to generalize better than dense models.

In order to get proper estimates of performance for models selected using RFE, this procedure was performed in a third, outer layer of 10-fold CV. This outer CV loop is used to define feature weights in the training set (9/10th of the data) and test the accuracy of the model in the validation set (1/10th of the data). Repetition of this procedure yields 10 models. The entire cross-validated RFE procedure was repeated 50 times, in order to further improve the performance estimate. The final prediction for a patient is an ensemble constituting the average of 50 repetitions.

Supplementary Note 4. Post hoc analyses

4.1 'Baseline' functional outcome prediction model

'Baseline' functional outcome prediction models were trained as follows: Functional status at baseline was used as the only feature to predict functional outcome at T3 and T6. We employed the same machine learning setup as for the full models, thus 50 iterations of SVM training using nested cross-validation, but without the RFE layer this time, since there is only one feature. These models resulted in mean (SD) balanced accuracies of 47.3% (2.3%) at T3 and 44.1% (1.6%) at T6. These single-feature models do not produce accurate predictions of functional outcome.

4.2 Using alternative definitions of good and poor functional outcome

To investigate the influence of the definition of good and poor outcome on the performance of our functional outcome prediction models, we applied different cut values to the GAF scores at T3 and T6. In our main approach, we used the cut value of 65. We retrained our models using a number of alternative GAF cut values: 50, 58 (mean value at baseline), 63/61 (producing about equally sized groups at T3/T6, respectively), and 68. Modeling was done in the same way as for the original models, see Table S15 below for the results. We found that a GAF cut of 65 yielded the highest balanced accuracy, while values around it yielded somewhat lower accuracies. The cut at 50 yielded quite lower accuracies (-10% or more as compared to the best accuracies) accompanied by large standard deviations. Thus, these results show that model performance does not very much depend on the definition of good and poor outcome for GAF cut values in the range 58-68, while lower GAF cuts lead to highly unbalanced groups with low numbers of patients in the poor outcome group, yielding prediction models with poorer performance.

4.3 Using an alternative learning design

For our modeling we have chosen to use linear support vector machine (SVM) in combination with recursive feature elimination (RFE). To investigate the possibility that the performance of our models is limited by the use of a linear classifier, we redid the prediction modeling on the full sample using a completely different setup: We used a random forests (RF) classifier, which is nonlinear by nature, in combination with IsoMap approach for computing a quasi-isometric and low-dimensional embedding of our initial raw features (instead of the RFE), as

implemented in the open source machine learning tool Neuropredict.[26] A 'light' grid search was used to optimize the hyper-parameters of RF on the training set. The results of the 50 iterations are shown in Table S16 below. As with the proposed method, we see that the performance for functional outcome is better than for symptomatic outcome. The mean balanced accuracy of the RF+IsoMap models is, averaged over the 8 different models, 2.6% lower than that of the proposed method (SVM+RFE). On the other hand, the area under the curve (AUC) is, on average, 0.04 higher for RF+IsoMap. The standard deviations of the proposed method are lower than those of RF+IsoMap (average values of 1.8% and 5.0%, respectively). From these results we conclude that the choice of a linear classification algorithm in combination with recursive feature elimination did not limit the performance of our models. From a qualitative point of view, the use of different learning algorithms may lead to models with different relationships between input features and output variables. Future studies with a more technical focus should further investigate the possibility of nonlinear effects of predictors on outcome.

4.4 Single-site prediction models as a probe of between-site differences

The leave-one-site-out (LOSO) models showed differences in performance between the four sites. Apart from ANOVAs to test for between-site differences at the level of individual predictors (see Supplemental Table S14), we also trained single-site models, and compared the resulting performances with those from the LOSO models. Table S17 below shows the results. The single-site results show larger variability in performance than the LOSO results, which is most probably due to the much lower sample sizes, possibly in combination with the samples being more homogeneous. Especially the Utrecht site, which had by far the lowest number of patients, showed lower single-site balanced accuracies for most models. On the other hand, the largest site, Maastricht, showed a slight increase in performance for most single-site models. Our conclusion is that the effect of between-site differences on the LOSO models is limited, and that predictions for a smaller site benefit from an increased robustness of models trained on the three other sites, as compared to the model trained on that site alone. Large enough sites may produce robust single-site models and benefit from the homogeneous sample.

Supplementary Table 1. Features per modality.

**Socio-demographic features T_0**
1. Age
2. Sex
3. Educational degree
4. Special education
5. Number of staying backs
6. Educational years
7. Socioeconomic status; educational level father
8. Socioeconomic status; educational degree father
9. Socioeconomic status; educational level mother
10. Socioeconomic status; educational degree mother
11. Number of moves
12. Subject has lost parent
13. Ethnicity
14. Household independent living
15. Subject has children
16. Employment
17. Student
**Illness related features T_0**
18. Illness duration
19. Frequency depression lifetime
20. Chronic course of illness
21. Episodic course of illness
22. One psychotic episode lifetime
23. Recent illness onset, in past year
24. Recent illness onset, in past two years
25. Quality of Life, health
26. Quality of Life
27. Duration untreated psychosis; first contact mental care institute
28. Duration untreated psychosis; start antipsychotic medication
29. Antipsychotic medication; current use
30. Antipsychotic medication; polytherapy
31. Clozapine current use
32. Suicide attempt lifetime
33. Diagnosis schizophrenia/psychosis related disorders
34. Global Assessment of Functioning; global functioning

35. Global Assessment of Functioning; degree of disabilities
36. Early age of onset, <19 years old
37. Normal age of onset, 20-30 years old
38. Late age of onset, >30 years old
**Positive and Negative Syndrome Scale T_0**
39. Delusions
40. Conceptual disorganization
41. Hallucinatory behavior
42. Excitement
43. Grandiosity
44. Suspiciousness/persecution
45. Hostility
46. Flat affect
47. Emotional withdrawal
48. Poor rapport
49. Passive/Apathetic Social withdrawal
50. Difficulty in abstract thinking
51. Lack of spontaneity
52. Stereotyped thinking
53. Somatic concern
54. Anxiety
55. Guilt feelings
56. Tension
57. Mannerism and posturing
58. Depression
59. Motor retardation
60. Lack of cooperation
61. Unusual thought content
62. Disorientation
63. Poor Attention
64. Poor Judgement and Insight
65. Avolition
66. Poor Impulse control
67. Preoccupation
68. Active social avoidance
**Substance use T_0**
69. Cannabis abuse/dependence and positive urinalysis
70. Cannabis abuse/dependence lifetime

71. Other illicit drug use present state
72. Other illicit drug use lifetime
73. Amount daily cigarettes (range 0-70)
74. Amount of weekly alcoholic units (range 0-70)
**Neurocognition T_0**
75. Wechsler Adult Intelligence Scale; digit symbol substitution (scaled score)
76. Wechsler Adult Intelligence Scale; block design (scaled score)
77. Wechsler Adult Intelligence Scale; calculation (scaled score)
78. Wechsler Adult Intelligence Scale; information (scaled score)
79. Continuous Performance Task; reaction time hits
80. Continuous Performance Task; number of false positives
81. Continuous Performance Task; number of false negative
82. Continuous Performance Task; number of correct positives
83. Response Shifting Task; accuracy cost score
84. Word Learning Task, 15 words; immediate recall
85. Word Learning Task, 15 words; delayed recall
**Social cognition T_0**
86. Hints total score
87. Benton Facial Recognition
88. Degraded Facial Affect Recognition; Neutral faces, amount correct
89. Degraded Facial Affect Recognition; Happy faces, amount correct
90. Degraded Facial Affect Recognition; Fearful faces, amount correct
91. Degraded Facial Affect Recognition; Angry faces, amount correct
**Premorbid Adjustment Scale (retrospective assessment, measured at T_0)**
92. Social Behavior <12 years old
93. Social Behavior 12-16 years old
94. Social Behavior 16-19 years old
95. Friendship <12 years old
96. Friendship 12-16 years old
97. Friendship 16-19 years old
98. School performance <12 years old
99. School performance 12-16 years old
100. School performance 16-19 years old
101. School adaptation <12 years old
102. School adaptation 12-16 years old
103. School adaptation 16-19 years old
104. Social sexual aspects 12-16 years old

105. Social sexual aspects 16-19 years old
106. Independence 16-19 years old
107. Highest level of functioning 16-19 years old
108. Social personal adaptation 16-19 years old
109. Interest in life 16-19 years old
110. Energy level 16-19 years old
**Camberwell Assessment of Needs Short Appraisal (CANSAS) T_0**
111. Number of no need
112. Number of unmet needs
113. Number of met needs
114. Housing need
115. Housing unmet need
116. Food need
117. Food need unmet need
118. Household need
119. Household unmet need
120. Self-care need
121. Self-care unmet need
122. Day time activities need
123. Day time activities unmet need
124. Physical health need
125. Physical health unmet need
126. Psychotic disorder need
127. Psychotic disorder unmet need
128. Information need
129. Information unmet need
130. Psychological distress need
131. Psychological distress unmet need
132. Safety to self need
133. Safety to self unmet need
134. Safety to others need
135. Safety to others unmet need
136. Alcohol need
137. Alcohol unmet need
138. Drugs need
139. Drugs unmet need
140. Company need
141. Company unmet need

142. Intimate relationships need
143. Intimate relationships unmet need
144. Sexual expression need
145. Sexual expression unmet need
146. Childcare need
147. Childcare unmet need
148. Education need
149. Education unmet need
150. Telephone need
151. Telephone unmet need
152. Transport need
153. Transport unmet need
154. Money need
155. Money unmet need
156. Welfare benefits need
157. Welfare benefits unmet need
158. Work need
159. Work unmet need
160. Side effects medication need
161. Side effects medication unmet need
**Community Assessment of Psychic Experiences T_0**
162. Feeling Sad
163. Feeling Sad- Distress
164. Other people say things with double meaning
165. Other people say things with double meaning- Distress
166. Lack of enthusiasm
167. Lack of enthusiasm- Distress
168. Not talkative when with other people
169. Not talkative when with other people- Distress
170. Messages on TV have special meaning
171. Messages on TV have special meaning- Distress
172. People are not what they seem (false appearance)
173. People are not what they seem (false appearance)- Distress
174. Persecution
175. Persecution- Distress
176. Lack of emotions
177. Lack of emotions- Distress
178. Feeling pessimistic

179. Feeling pessimistic- Distress
180. Conspiracy
181. Conspiracy- Distress
182. Important person
183. Important person- Distress
184. No future
185. No future- Distress
186. Special person
187. Special person- Distress
188. Suicidal
189. Suicidal- Distress
190. Telepathy
191. Telepathy- Distress
192. No interest in others
193. No interest in others- Distress
194. Influenced by devices
195. Influenced by devices- Distress
196. Lack of motivation
197. Lack of motivation- Distress
198. Crying
199. Crying- Distress
200. Voodoo
201. Voodoo- Distress
202. Lack of energy
203. Lack of energy- Distress
204. Odd look
205. Odd look- Distress
206. Empty mind
207. Empty mind- Distress
208. Thought withdrawal
209. Thought withdrawal- Distress
210. Lack of activity
211. Lack of activity- Distress
212. Thought insertion
213. Thought insertion- distress
214. Blunted affect
215. Blunted affect- Distress
216. Blunted emotions

217. Blunted emotions- Distress
218. Thought broadcasting
219. Thought broadcasting- Distress
220. Lack of spontaneity
221. Lack of spontaneity- Distress
222. Thought echo
223. Thought echo- Distress
224. External control
225. External control- Distress
226. Hallucinations
227. Hallucinations- Distress
228. Voices conversing
229. Voices conversing- Distress
230. Lack of personal hygiene
231. Lack of personal hygiene- Distress
232. Unable to terminate
233. Unable to terminate- Distress
234. Lack of hobbies
235. Lack of hobbies- Distress
236. Guilty
237. Guilty- Distress
238. Failure
239. Failure- Distress
240. Feeling tense
241. Feeling tense- Distress
242. Capgras
243. Capgras- Distress
244. Visual hallucinations
245. Visual hallucinations- Distress
**Extrapyramidal symptoms T_0**
246. Akathisia
247. Dystonia
248. Abnormal Involuntary Movement rating Scale
249. Unified Parkinson Disease Rating Scale
**Genetic characteristics T_0**
250. Polygenic risk score (PRS threshold p=0.1)
251. Familial loading; psychotic disorder
252. Familial loading; bipolar disorder

253. Familial loading; drug abuse
**Environmental T_0**
254. Number of people living with the patient
255. Maltreatment/assault before psychosis
256. Maltreatment/assault after psychosis
257. Urbanicity at birth
258. Ubanicity current
**Set of predictors of four-week Global Assessment of Functioning-based outcome European First Episode Schizophrenia Trial (EUFEST)**
1. Employment
2. Student
3. Daytime activities need (CANSAS)
4. Daytime activities unmet need (CANSAS)
5. Psychological distress need (CANSAS)
6. Psychological distress unmet need (CANSAS)
7. Company need (CANSAS)
8. Company unmet need (CANSAS)
9. Money need (CANSAS)
10. Money unmet need (CANSAS)
11. Global Assessment of Functioning, global functioning
12. Global Assessment of Functioning, degree of disabilities
13. Total Positive and Negative Symptom Scale symptom severity score
14. Sum of no-need items (CANSAS)
15. Education of mother, highest level
16. Educational years patient (excluding staying backs)
17. Sum of unmet-need items (CANSAS)
18. Information need (CANSAS)
19. Information unmet need (CANSAS)
20. Present diagnosis of schizophrenia
21. Accommodation need (CANSAS)
22. Accommodation unmet need (CANSAS)
23. Sexual expression need (CANSAS)
24. Sexual expression unmet need (CANSAS)
**Set of predictors of 52-week Global Assessment of Functioning-based outcome EUFEST**
1. Employment
2. Student
3. Company need (CANSAS)

4. Company unmet need (CANSAS)
5. PANSS P04: hyperactivity
6. Daytime activities need (CANSAS)
7. Daytime activities unmet need (CANSAS)
8. Psychological distress need (CANSAS)
9. Psychological distress unmet need (CANSAS)
10. Sex
11. PANSS positive score
12. PANSS P02: conceptual disorganization
13. Suicide attempt lifetime
14. Global Assessment of Functioning, global functioning
15. Global Assessment of Functioning, degree of disabilities
16. Safety to others need (CANSAS)
17. Safety to others unmet need (CANSAS)
18. Present diagnosis of schizophrenia
19. Number of needs (CANSAS)
20. Special education
21. Number of staying backs
22. Sum of unmet-need items (CANSAS)

Supplementary Table 2. Summary of the number of features in each unimodal predictor modality, sample size per modality and per outcome, and distributions of good versus poor symptomatic and global outcome.

**Modalities (T_0)**	**No. of fea-tures**	***n* Symptomatic outcome [a] T_3**	**% poor symptomatic outcome T_3**	***n* Symptomatic outcome T_6**	**% poor symptomatic outcome T_6**	***n* Global outcome [b] T_3**	**% poor global outcome T_3**	***n* Global outcome T_6**	**% poor global outcome T_6**
Demographic	18	523	63%	523	59%	442	56%	484	64%
Illness related	20	523	63%	523	59%	442	56%	484	64%
PANSS	30	523	63%	523	59%	442	56%	484	64%
Substance use	6	523	63%	523	59%	442	56%	484	64%
Neurocognition	11	437	63%	437	61%	368	56%	404	65%
Social cognition	6	437	63%	437	61%	368	56%	404	65%
PAS	19	422	62%	422	58%	389	55%	427	63%
CANSAS	51	332	62%	332	60%	274	54%	313	62%
CAPE	84	445	63%	445	58%	377	56%	414	65%
EPS	4	506	62%	506	59%	426	56%	468	63%
Genetic	4	291	62%	291	58%	240	54%	268	63%
Environmental	5	280	67%	280	60%	237	59%	270	66%

[a] Symptomatic outcome based on remission criteria adapted from Andreasen *et al.* (2005).

[b] Global outcome based on GAF; American Psychiatric Association (2000).

Supplementary Table 3. Summary of the total number of features in the multimodel models, sample size per model and per outcome, and distributions of good versus poor symptomatic (remission) and global (GAF) outcome.

**Modalities (T_0)**	**No. of features**	***n* Symptomatic outcome T_3**	**% poor symptomatic outcome T_3**	***n* Symptomatic outcome T_6**	**% poor symptomatic outcome T_6**	***n* Global outcome T_3**	**% poor global outcome T_3**	***n* Global outcome T_6**	**% poor global outcome T_6**
PANSS, ill, demo, CANSAS	119	332	62%	332	60%	274	54%	313	62%
PANSS, ill, demo, CAPE	152	445	63%	445	58%	377	56%	414	65%
EUFEST 4 weeks	24	332	62%	332	60%	274	54%	313	65%
EUFEST 52 weeks	22	332	62%	332	60%	274	54%	313	65%

EUFEST 4 weeks is set of 10% best performing features of four-week outcome prediction of the European First Episode Schizophrenia Trial; EUFEST 52 weeks is set of 10% best performing features of 52-week outcome prediction of the European First Episode Schizophrenia Trial.

Supplementary Table 4. Results of linear nested cross-validated models, of T_0 GROUP features as predictors, classifying schizophrenia patients in good versus poor symptomatic outcome and good versus poor global outcome.

**Feature Modalities (T_0)**	**Sens Symptomatic outcome T_3**	**Spec Symptomatic outcome T_3**	**BAC Symptomatic outcome T_3**	**PPV/NPV Symptomatic outcome T_3**	**Sens Symptomatic outcome T_6**	**Spec Symptomatic outcome T_6**	**BAC Symptomatic outcome T_6**	**PPV/NPV Symptomatic outcome T_6**
Demographics	55.2	61.1	58.2	70.1/44.7	55.9	59.2	57.6	64.8/46.5
Illness related	62.3	65.8	64.1	77.0/52.4	65.8	53.6	59.7	65.5/49.8
PANSS	54.4	71.9	63.1	77.8/49.1	69.3	58.0	63.7	72.7/51.4
Substance use	48.1	58.6	53.3	64.7/38.9	54.9	43.1	49.0	52.2/31.7
Neurocognition	53.6	54.5	54.1	66.1/39.7	56.6	54.4	55.5	63.0/40.6
Social cognition	44.1	48.3	46.2	53.9/28.8	38.8	64.4	51.6	57.7/36.5
PAS	53.4	56.8	55.1	65.8/41.8	49.6	64.0	56.8	58.8/43.2
CANSAS	53.2	59.1	56.1	69.2/44.3	57.8	60.5	59.1	67.4/47.5
CAPE	55.4	55.0	55.2	67.5/41.6	52.2	55.5	53.9	63.1/46.5
EPS	34.9	73.9	54.4	69.5/40.6	38.0	72.8	55.4	67.6/45.8
Genetics	43.2	64.2	53.7	56.9/35.1	36.3	61.2	48.8	47.7/36.1
Environmental	47.2	61.4	54.3	74.4/38.1	30.0	70.7	50.4	52.3/36.1
	**Sens Global outcome T_3**	**Spec Global outcome T_3**	**BAC Global outcome T_3**	**PPV/NPV Global outcome T_3**	**Sens Global outcome T_6**	**Spec Global outcome T_6**	**BAC Global outcome T_6**	**PPV/NPV Global outcome T_6**
Demographics	60.6	61.6	61.1	65.3/53.9	60.1	62.1	61.1	74.8/52.0
Illness related	66.3	58.8	62.6	68.3/59.3	69.8	57.2	63.5	74.8/52.0
PANSS	61.2	67.5	64.3	72.0/59.2	55.7	68.4	62.0	77.5/48.2
Substance use	45.1	67.1	56.1	64.0/49.3	53.8	53.4	53.6	65.2/37.6

Neurocognition	59.3	50.5	54.9	58.3/47.3	59.1	54.6	56.9	70.0/41.7
Social cognition	54.8	41.1	47.9	51.5/39.0	46.7	62.8	54.8	68.0/38.0
PAS	48.8	62.9	55.9	61.3/50.0	53.3	62.1	57.7	69.6/42.6
CANSAS	61.5	58.6	60.1	3.3//56.3	59.2	62.3	60.8	69.9/46.7
CAPE	59.7	58.2	59.0	64.8/52.5	52.8	58.8	55.8	70.4/40.3
EPS	30.6	81.0	55.8	68.3/48.1	33.7	78.7	56.2	70.7/40.3
Genetics	41.9	68.5	55.2	68.3/48.1	51.8	65.8	58.8	68.3/40.8
Environmental	42.4	42.2	42.3	44.9/27.7	42.3	53.7	48.0	63.9/31.8

Supplementary Table 5. Prediction of symptomatic outcome at T_3 with predictors of PANSS, demographic, illness related and CANSAS.

Feature	psel	beta	beta S.D.
PANSS Poor Judgement and Insight	1.00	0.47	0.35
CANSAS housing need	1.00	0.35	0.11
PANSS Hallucinatory behavior	0.99	0.18	0.18
DEMO Age	0.99	0.50	0.46
ILL GAF disabilities	0.99	-0.22	0.20
ILL Diagnosis schizophrenia/psychosis related disorders	0.98	-0.28	0.11
CANSAS number of no need	0.96	0.03	0.16
ILL Quality of Life health	0.94	-0.46	0.37
PANSS Suspiciousness/persecution	0.92	0.37	0.31
CANSAS number of met needs	0.90	-0.04	0.13
ILL duration of illness	0.80	0.05	0.09
DEMO Educational degree	0.76	0.04	0.11

Supplementary Table 6. Prediction of symptomatic outcome at T_3 with predictors of PANSS, demographic, illness related and CAPE.

Feature	psel	beta	beta S.D.
PANSS Hallucinatory behavior	1.00	0.29	0.17
ILL Diagnosis schizophrenia/psychosis related disorders	1.00	-0.45	0.12
ILL GAF disabilities	1.00	-0.43	0.26
CAPE Guilty	1.00	-0.15	0.16
PANSS Poor Judgement and Insight	1.00	0.51	0.27
ILL GAF symptoms	0.98	-0.12	0.12
DEMO Age	0.96	0.45	0.45
CAPE Suicidal	0.92	0.35	0.27
ILL Quality of Life health	0.91	-0.38	0.25
CAPE Guilty- Distress	0.89	-0.35	0.20
PANSS Suspiciousness/persecution	0.88	0.19	0.15
PANSS Lack of spontaneity	0.84	0.19	0.18
PANSS Excitement	0.79	0.28	0.27
ILL Quality of Life	0.79	-0.09	0.12
PANSS Depression	0.75	0.25	0.23

Supplementary Table 7. Prediction of symptomatic outcome at T6, with predictors of PANSS, demographic, illness related and CANSAS.

Feature	psel	beta	beta S.D.
ILL Status Antipsychotics	1.00	0.48	0.15
ILL GAF symptoms	1.00	-0.29	0.21
PANSS Delusions	0.99	0.29	0.19
PANSS Poor Judgement and Insight	0.99	0.54	0.38
DEMO Socioeconomic status; educational degree father	0.98	-0.32	0.19
ILL Diagnosis schizophrenia/psychosis related disorders	0.98	-0.20	0.08
DEMO Subject has children	0.96	-0.36	0.14
ILL GAF disabilities	0.93	0.10	0.20
CANSAS food need	0.92	-0.35	0.16
CANSAS psychotic disorder unmet need	0.92	0.25	0.11
DEMO Socioeconomic status; educational degree mother	0.91	-0.27	0.22
PANSS Flat affect	0.90	0.25	0.24

Supplementary Table 8. Prediction of symptomatic outcome at T_6 with predictors of PANSS, demographic, illness related and CAPE.

Feature	psel	beta	beta S.D.
ILL GAF symptoms	1.00	-0.53	0.34
ILL GAF disabilities	1.00	-0.27	0.19
PANSS Unusual thought content	0.99	0.22	0.14
PANSS Hallucinatory behavior	0.99	0.26	0.17
PANSS Emotional withdrawal	0.97	0.25	0.20
PANSS Delusions	0.95	0.31	0.16
CAPE Lack of activity- Distress	0.93	0.13	0.13
PANSS Flat affect	0.93	0.23	0.16
ILL Status Antipsychotics	0.92	0.29	0.12
CAPE Hallucinations	0.91	0.19	0.13
PANSS Lack of spontaneity	0.91	0.15	0.14
PANSS Poor Judgement and Insight	0.90	0.24	0.18
PANSS Motor retardation	0.88	0.17	0.14
CAPE Lack of activity	0.87	0.25	0.19
PANSS Difficulty in abstract thinking	0.87	0.23	0.22

Supplementary Table 9. Prediction of global outcome at T_3 with predictors of PANSS, demographic, illness related and CANSAS.

Feature	psel	beta	beta S.D.
ILL GAF symptoms	1.00	-0.23	0.19
PANSS Stereotyped thinking	1.00	0.73	0.46
ILL GAF disabilities	1.00	-0.28	0.26
CANSAS number of no need	1.00	-0.15	0.27
PANSS Hallucinatory behavior	0.99	0.67	0.37
CANSAS number of met needs	0.93	-0.21	0.23
PANSS Motor retardation	0.88	0.36	0.29
PANSS Unusual thought content	0.86	0.27	0.22
PANSS Flat affect	0.84	0.38	0.31
PANSS Passive/Apathetic Social withdrawal	0.81	0.45	0.38
CANSAS housing need	0.81	0.18	0.13
CANSAS food need	0.74	0.13	0.12

Supplementary Table 10. Prediction of global outcome at T_3 with predictors of PANSS, demographic, illness related and CAPE.

Feature	psel	beta	beta S.D.
ILL GAF symptoms	1.00	-0.58	0.22
ILL GAF disabilities	1.00	-0.68	0.27
PANSS Stereotyped thinking	1.00	0.47	0.21
PANSS Motor retardation	1.00	0.63	0.25
PANSS Passive/Apathetic Social withdrawal	0.99	0.74	0.30
PANSS Unusual thought content	0.98	0.34	0.18
PANSS Flat affect	0.95	0.42	0.18
PANSS Difficulty in abstract thinking	0.92	0.45	0.26
PANSS Poor Judgement and Insight	0.92	0.35	0.17
PANSS Conceptual disorganization	0.91	0.27	0.16
ILL Quality of Life	0.90	-0.41	0.23
PANSS Grandiosity	0.84	0.38	0.26
CAPE Feeling tense- Distress	0.81	0.40	0.25
PANSS Emotional withdrawal	0.79	0.22	0.16
DEMO Educational degree	0.76	0.01	0.16

Supplementary Table 11. Prediction of global outcome at T_6 with predictors of PANSS, demographic, illness related and CANSAS.

Feature	psel	beta	beta S.D.
ILL GAF symptoms	1.00	-0.14	0.21
ILL GAF disabilities	1.00	-0.46	0.37
CANSAS number of no need	1.00	-0.05	0.20
CANSAS number of met needs	1.00	-0.11	0.25
CANSAS housing need	0.96	0.24	0.09
PANSS Hallucinatory behavior	0.95	0.41	0.29
ILL Quality of Life	0.95	-0.45	0.37
PANSS Poor Judgement and Insight	0.94	0.22	0.18
PANSS Tension	0.92	0.23	0.24
ILL Status Antipsychotics	0.89	0.32	0.16
CANSAS number of unmet needs	0.83	-0.08	0.22
CANSAS day time activities need	0.83	0.06	0.07

Supplementary Table 12. Prediction of global outcome at T_6 with predictors of PANSS, demographic, illness related and CAPE.

Feature	psel	beta	beta S.D.
ILL GAF symptoms	1.00	-0.32	0.18
ILL GAF disabilities	1.00	-0.89	0.44
DEMO Employment	0.99	-0.28	0.07
PANSS Unusual thought content	0.98	0.19	0.18
ILL Quality of Life	0.98	-0.42	0.21
PANSS Grandiosity	0.97	0.81	0.38
PANSS Tension	0.92	0.57	0.34
PANSS Motor retardation	0.89	0.16	0.19
ILL Quality of Life health	0.89	-0.41	0.23
PANSS Flat affect	0.88	0.26	0.18
PANSS Depression	0.88	0.33	0.21
PANSS Poor Judgement and Insight	0.84	0.09	0.17
PANSS Passive/Apathetic Social withdrawal	0.81	0.19	0.18
PANSS Hallucinatory behavior	0.81	0.26	0.20
CAPE Telepathy	0.78	0.30	0.21

Supplementary Table 13. Differences in sociodemographic and clinical characteristics between patients with large proportion of false negatives versus those with a large proportion of true positives; and between patients with large proportion of false positives versus those with a large proportion of true negatives.

	Symptomatic outcome								Global outcome							
	T_3				T_6				T_3				T_6			
	CANSAS		CAPE		CANSAS		CAPE		CANSAS		CAPE		CANSAS		CAPE	
	FN-TP	FP-TN	FN-TP	FP-TN	FN-TP	FP-TN	FN-TP	FP-TN	FN-TP	FP-TN	FN-TP	FP-TN	FN-TP	FP-TN	FN-TP	FP-TN
Age (years; mean±S.D.)	↓	-	↓	-	-	-	-	-	-	↓	-	-	-	-	-	-
Sex (% male)	-	↑	-	-	↓	-	↓	-	-	↑	-	-	↓	-	-	-
Ethnicity (% white)	-	-	-	-	-	-	-	-	-	-	-	-	-	-	-	-
WAIS IQ (mean±S.D.)	↑	↓	↑	-	-	↓	-	↓	s	-	-	-	-	-	-	-
Hinting task (TOM)	-	-	-	-	-	-	-	-	↑	-	↑	-	-	-	-	-
Education patient (mean±S.D.)	-	-	-	↓	-	-	-	↓	↑	-	↑	-	-	↓	-	-
Socioeconomic status, education father (mean±S.D.)	-	-	-	-	↑	-	-	-	↑	↑	↑	-	↑	-	-	-
Socioeconomic status, education mother (mean±S.D.)	↑	-	↑	-	-	-	-	-	-	-	-	-	-	-	-	-
Employment/student (% yes)	-	-	-	↑	-	-	-	-	-	-	-	-	-	↑	-	-
Age of onset (years; mean±S.D.)	↓	-	↓	-	-	-	-	-	-	-	-	-	-	-	-	-
DSM-IV schizophrenia diagnosis (% 295.1.2.3)	↓	↑	↓	-	↓	-	↓	↑	-	↑	-	↑	↓	-	↓	↑
Clozapine current use	-	-	-	-	-	↑	-	-	-	↑	-	↑	-	-	-	-
PANSS positive symptoms (mean±S.D.)	↓	↑	↓	↑	-	↑	↓	↑	↓	↑	↓	↑	↓	↑	↓	↑
PANSS negative symptoms (mean±S.D.)	↓	↑	↓	↑	↓	↑	↓	↑	↓	↑	↓	↑	-	↑	↓	↑
PANSS general symptoms (mean±S.D.)	↓	↑	↓	↑	↓	↑	↓	↑	↓	↑	↓	↑	-	↑	↓	↑
Recent onset of psychosis 12 months	-	-	-	-	-	-	-	↑	-	-	-	-	-	-	-	-
Recent onset of psychosis 24 months	-	-	-	-	-	-	-	-	-	-	-	-	-	-	-	-
Global Assessment of Functioning; global functioning	↑	↓	↑	↓	↑	↓	↑	↓	↑	↓	↑	↓	↑	↓	↑	↓
Global Assessment of Functioning; degree of disabilities	↑	↓	↑	↓	↑	↓	↑	↓	↑	↓	↑	↓	↑	↓	↑	↓
Premorbid Adjustment Scale overall score	↓	↑	↓	-	-	-	-	↑	↓	↑	↓	↑	-	↑	-	↑
Chronicity	↓	↑	↓	↑	-	-	-	↑	↓	↑	↓	↑	-	↑	-	↑

A positive outcome indicates a poor outcome; a negative outcome indicates a good outcome. Higher score; significant at $p<0.05$; ↓: lower score; significant at $p<0.05$; - no significant difference; FN-TP is the comparison between those with ≥90% false negatives and those who have ≥90% true positives; FP-TN is the comparison between those with ≥90% false positives and those who have ≥90% true negatives. Abbreviations for table S1-S13: T_0 is baseline measurement; T_3 is follow-up at three-years interval after the baseline; T_6 is follow-up at six-years interval after the baseline; rem is remission; GAF is global assessment of functioning; sens is sensitivity; spec is specificity; BAC is balanced accuracy; PPV is positive predictive value; NPV is negative predictive value; PANSS is Positive and Negative Syndrome Scale; CANSAS is Camberwell Assessment of Needs Short Appraisal; ILL is illness related feature; DEMO is demographic feature; CAPE is community assessment of psychic experiences. PAS is premorbid adjustment scale; EPS is extrapyramidal symptom; WAIS is Wechsler adult intelligence scale; TOM is Theory of Mind; DSM is Statistical Manual of Mental Disorders.

Supplementary Table 14: between-site differences for Socio-demographic and illness related features, PANSS, CANSAS and CAPE

	**Amsterdam[1]**	**Utrecht[1]**	**Groningen[1]**	**Maastricht[1]**	**X^2 / F [2]**	**p**	**Post-hoc[3]**
**Socio-demographic features T_0**							
Age	29.06 (8.17)	26.88 (6.05)	27.05 (6.77)	27.34 (8.07)	2.370	0.070	
Sex (male)[a]	111 (82.2)	80 (85.1)	114 (77.0)	97 (66.4)	14.697	0.002	
Educational degree	4.48 (2.14)	3.85 (1.92)	4.09 (1.87)	4.53 (1.87)	3.247	0.022	
Special education							
Number of staying backs	0.98 (1.07)	0.79 (0.80)	0.82 (0.89)	1.05 (0.97)	2.335	0.073	
Educational years	11.50 (2.67)	10.66 (2.28)	10.82 (2.26)	11.47 (2.35)	4.103	0.007	
Socioeconomic status; educational level father	5.61 (2.44)	4.90 (2.48)	4.69 (2.54)	5.24 (2.27)	3.699	0.12	
Socioeconomic status; educational degree mother	4.39 (2.54)	4.61 (2.11)	3.99 (2.24)	4.82 (2.24)	3.313	0.20	
Number of moves	3.14 (2.50)	2.82 (2.40)	3.26 (2.31)	3.26 (2.34)	2.517	0.057	
Subject has lost parent (yes)[a]	24 (18.2)	10 (10.6)	18 (12.2)	19 (13.0)	3.351	0.341	
Ethnicity (white)[a]	113 (83.7)	81 (86.2)	123 (83.1)	132 (90.4)	3.936	0.268	
Household independent living (yes)[a]	62 (45.9)	42 (44.7)	68 (45.9)	64 (43.8)	0.181	0.981	
Subject has children (yes)[a]	10 (7.4)	4 (4.3)	12 (8.1)	15 (10.3)	2.919	0.404	
Employment (yes)[a]	47 (34.8)	31 (33.0)	50 (33.8)	52 (35.6)	0.215	0.975	
Student (yes)[a]	17 (12.6)	11 (11.7)	13 (8.8)	20 (13.7)	1.891	0.595	
**Illness related features T_0**							
Illness duration	4.73 (5.02)	4.54 (3.87)	4.46 (4.42)	5.57 (4.16)	1.776	0.959	

Frequency depression lifetime							
Chronic course of illness (yes)[a]	116 (85.9)	77 (81.9)	116 (78.4)	127 (87.0)	4.815	0.186	
Episodic course of illness (yes)[a]	79 (58.5)	61 (64.9)	103 (69.6)	69 (47.3)	16.539	0.001	
Recent illness onset, in past year (yes)[a]	30 (22.2)	29 (30.9)	36 (24.3)	15 (10.3)	16.711	0.001	
Recent illness onset, in past two years (yes)[a]	42 (31.1)	38 (40.4)	53 (35.8)	35 (24.7)	7.658	0.054	
Quality of Life, health	3.26 (1.12)	3.18 (1.08)	3.52 (1.07)	3.55 (0.99)	3.380	0.018	
Quality of Life	3.50 (0.99)	3.22 (1.01)	3.47 (0.94)	3.71 (0.93)	4.407	0.005	U<M
Duration untreated psychosis; first contact mental care institute	1.35 (3.91)	0.40 (1.19)	0.81 (1.94)	0.43 (1.30)	4.371	0.005	A>U; A>G; A>M
Duration untreated psychosis; start antipsychotic medication	1.28 (3.61)	0.66 (2.08)	0.94 (2.08)	0.62 (1.39)	2.154	0.093	
Antipsychotic medication; current use (yes)[a]	124 (91.9)	86 (91.5)	141 (95.1)	128 (87.7)	5.641	0.137	
Antipsychotic medication; polytherapy (yes)	6 (4.4)	9 (9.6)	16 (10.8)	18 (12.3)	5.729	0.126	
Clozapine current use (yes)[a]	13 (9.6)	22 (23.4)	17 (11.5)	12 (8.2)	14.042	0.003	
Suicide attempt lifetime (yes)[a]	31 (23.0)	20 (21.3)	37 (25.0)	40 (27.4)	1.384	0.709	
Diagnosis schizophrenia (yes)[a]	91 (67.4)	66 (70.2	90 (60.8)	95 (65.1)	2.587	0.460	
Global Assessment of Functioning; global functioning	55.76 (17.08)	51.73 (14.90)	62.24 (13.76)	59.50 (16.40)	10.049	<0.001	U<G; U<M
Global Assessment of Functioning; degree of disabilities	55.14 (15.21)	52.32 (15.12)	59.18 (14.36)	59.68 (16.50)	6.064	<0.001	U<G; U<M
Early age of onset, <19 years old (yes)[a]	41 (30.4)	27 (28.7)	46 (31.1	53 (36.3)	1.928	0.587	
Normal age of onset, 20-30 years old (yes)	70 (51.9)	57 (60.6)	87 (58.8)	73 (50.0)	4.074	0.254	
Late age of onset, >30 years old (yes)[a]	24 (17.8)	10 (10.6)	15 (10.1)	19 (13.0)	4.262	0.235	

**Positive and Negative Syndrome Scale (PANSS) T_0**							
Positive subscale	11.60 (4.93)	15.18 (5.67)	11.22 (4.30)	11.77 (4.99)	14.449	<0.001	A<U; G<U; M<U
Negative subscale	14.83 (6.10)	15.81 (5.51)	12.81 (4.46)	10.98 (5.05)	20.783	<0.001	A>G; A>M; G<U; G>M; U>M
General subscale	26.46 (7.20)	30.85 (8.68)	26.67 (7.28)	25.35 (7.33)	10.773	<0.001	A<U; G<U; M<U
**Camberwell Assessment of Needs Short Appraisal (CANSAS) T_0**							
Number of needs	6.32 (3,59)	8.20 (4.30)	6.55 (3.77)	6.28 (3.74)	3.594	0.014	U>A; U>M
Number of no need	17.68 (3.59)	15.79 (4.31)	17.45 (3.77)	17.34 (3.86)	3.594	0.014	U<A; U<M
Number of unmet needs	2.85 (2.49)	4.17 (2.87)	2.74 (3.06)	1.99 (2.27)	8.784	<0.001	U>A; U>G
Number of met needs	3.47 (2.82)	4.03 (3.03)	3.81 (2.46)	4.30 (3.20)	1.355	0.257	
**Community Assessment of Psychic Experiences (CAPE) T_0**							
Positive symptoms (frequency)	0.64 (0.44)	0.69 (0.53)	0.65 (0.46)	0.68 (0.55)	0.415	0.742	
Positive symptoms (distress)	1.27 (0.65)	1.21 (0.76)	1.31 (0.67)	1.30 (0.68)	0.448	0.506	
Negative symptoms (frequency)	0.98 (0.53)	1.05 (0.61)	0.99 (0.54)	1.02 (0.55)	0.779	0.817	
Negative symptoms (distress)	1.28 (0.61)	1.29 (0.63)	1.27 (0.60)	1.28 (0.61)	0.046	0.719	
Depressive symptoms (frequency)	0.96 (0.54)	0.99 (0.60)	1.03 (0.63)	1.01 (0.59)	0.312	0.987	
Depressive symptoms (distress)	1.45 (0.69)	1.42 (0.74)	1.55 (0.64)	1.48 (0.68)	0.851	0.466	

[a]factor (without [a] : continuous measure)

[1]mean (Standard Deviation) reported for continuous measures, number (%) for factors.

[2]F-test (F) for continuous measures with post-hoc pairwise comparisons in case of significant ($p<0.05$ level) differences for continuous measures, Chi-squared (χ) for factors.

[3]significant (bonferoni corrected within item, at 0.05 level) post-hoc comparisons. A= Amsterdam; G= Groningen; M= Maastricht; U= Utrecht.

Supplementary Table 15. Full-sample cross-validation performance, based on good/poor outcome definitions with different GAF cut values.

GAF cut value	N(good)/N(poor) T3	BAC T3	N(good)/N(poor) T6	BAC T6
50	215/59	54.8 (10.1)	242/71	59.3 (5.3)
58	162/112	64.6 (2.4)	186/127	65.6 (2.0)
63/61*	132/142	63.0 (1.7)	159/154	63.2 (2.0)
65**	127/147	67.2 (1.9)	134/179	69.2 (2.2)
68	110/164	65.7 (2.2)	119/194	68.5 (2.0)

*For T3/T6, respectively. **Value used in our main approach. Abbreviations: BAC is balanced accuracy.

Supplementary Table 16. Full-sample cross-validation performance, using random forests classifier with IsoMap, implemented in Neuropredict.

**Predictor/Model (outcome)**	Internal BAC	Internal AUC
PANSS, ill, demo, CANSAS (symptomatic outcome T_3)	59.3 (5.7)	0.64 (0.07)
PANSS, ill, demo, CAPE (symptomatic outcome T_3)	56.9 (4.6)	0.62 (0.05)
PANSS, ill, demo, CANSAS (symptomatic outcome T_6)	62.5 (5.1)	0.67 (0.06)
PANSS , ill, demo, CAPE (symptomatic outcome T_6)	60.5 (4.3)	0.66 (0.06)
PANSS, ill, demo, CANSAS (global outcome T_3)	67.2 (4.7)	0.72 (0.06)
PANSS, ill, demo, CAPE (global outcome T_3)	62.3 (4.7)	0.67 (0.06)
PANSS, ill, demo, CANSAS (global outcome T_6)	69.2 (6.1)	0.74 (0.06)
PANSS, ill, demo, CAPE (global outcome T_6)	61.2 (4.7)	0.69 (0.06)

Abbreviations: BAC (mean (SD)) is balanced accuracy; AUC is area under the curve.

Supplementary Table 17. Single-site cross-validation performance, predicting symptomatic and global outcome at T_3 and T_6.

	N	BAC Symptomatic outcome T_3 [a]	N	BAC Symptomatic outcome T_3 [b]	N	BAC Symptomatic outcome T_6 [a]	N	BAC Symptomatic outcome T_6 [b]
Amsterdam	81	62.7 (4.5)	104	64.4 (4.4)	81	57.0 (6.8)	104	61.4 (6.1)
Groningen	73	62.1 (5.3)	132	58.6 (3.1)	73	62.7 (5.9)	132	55.2 (4.5)
Maastricht	124	59.3 (4.5)	139	52.7 (4.3)	124	65.9 (3.1)	139	68.3 (3.0)
Utrecht	54	41.1 (7.2)	70	49.8 (19.5)	54	42.2 (9.5)	70	46.7 (9.7)
	N	BAC Global outcome T_3 [a]	N	BAC Global outcome T_3 [b]	N	BAC Global outcome T_6 [a]	N	BAC Global outcome T_6 [b]
Amsterdam	80	57.9 (4.8)	100	61.3 (3.9)	77	59.4 (4.8)	98	56.9 (4.6)
Groningen	65	69.0 (5.1)	118	65.4 (3.6)	58	68.9 (7.6)	107	61.3 (4.6)
Maastricht	81	64.2 (4.6)	93	65.2 (4.3)	124	76.2 (3.6)	139	71.2 (4.0)
Utrecht	48	75.1 (5.3)	66	65.3 (13.9)	54	53.0 (10.5)	70	50.6 (12.3)

Rows mention the geographic site. Abbreviations: BAC (mean (SD)) is balanced accuracy; T_3 is follow-up at three-year interval after the baseline; T_6 is follow-up at six-year interval after the baseline.

[a] Models contained PANSS, demographic, illness and CANSAS features.

[b] Models contained PANSS, demographic, illness and CAPE features

Supplementary Figure 1. Selection process of the sample used in this study.

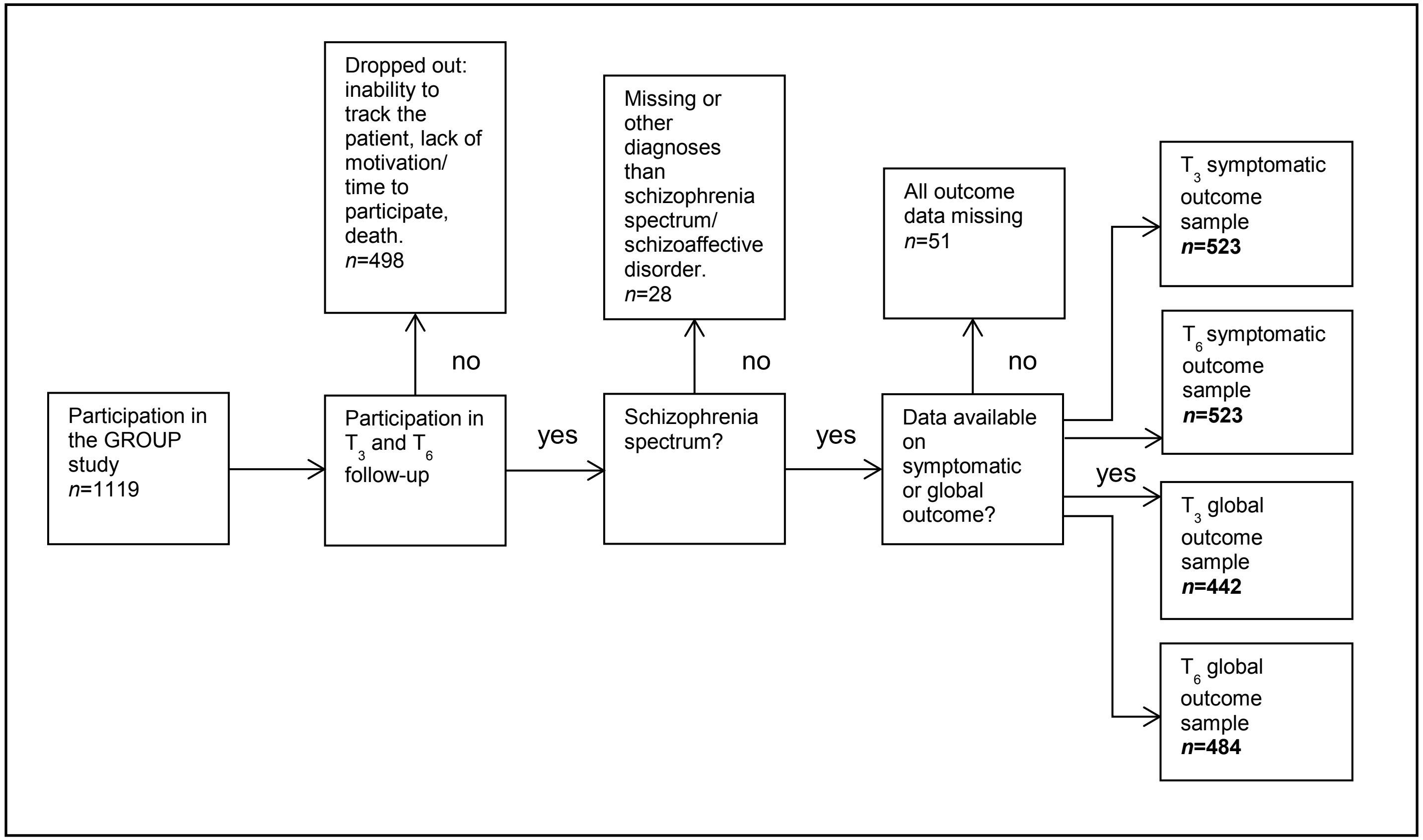

Supplementary Figure 2. Good/Poor outcome Ratios per timepoint and overview of longitudinal course within the sample based on good/poor outcome

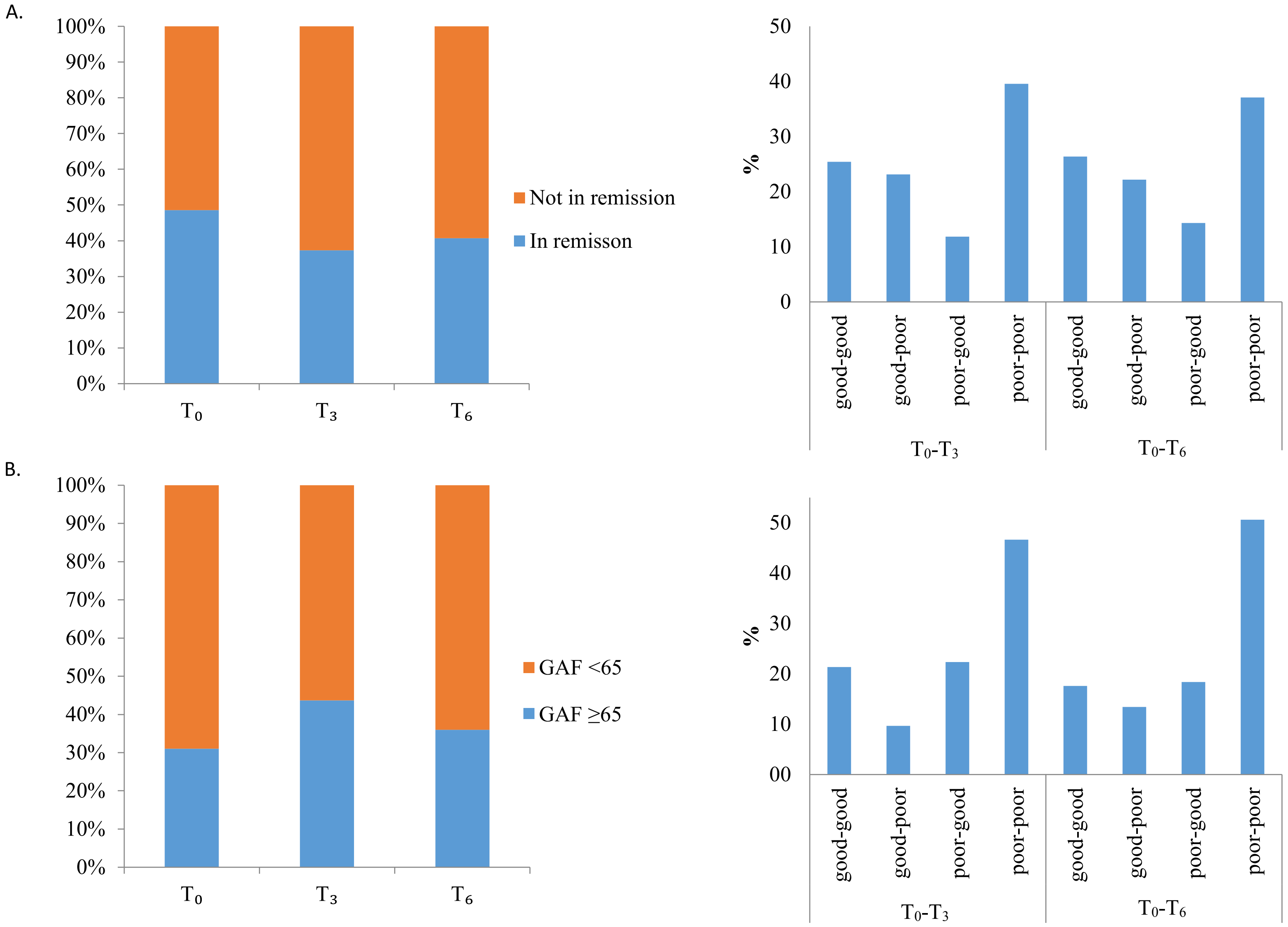

A. Ratios (left) and longitudinal course (right) of symptomatic outcome during baseline (T_0) compared to follow-up (T_3, three years after baseline; 'T_6, six years after baseline). T_0 remission was only available without time component, thus solely based on PANSS symptoms.

B. Ratios (left) and longitudinal course (right) of global outcome during baseline and follow-up.

'Good' means good outcome: in remission (A) or GAF ≥65 (B); 'poor' means poor outcome: not in remission (A) or GAF <65 (B).

Supplementary Figure 3. Frequency of inclusion of a feature against its (average) weight in the model

A. Symptomatic outcome T_3; PANSS, ill, demo, CANSAS

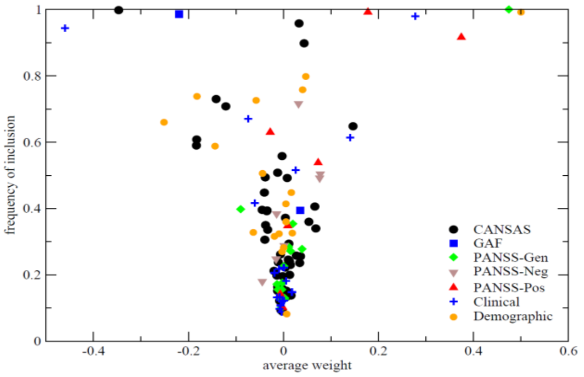

B. Symptomatic outcome T_3; PANSS, ill, demo, CAPE

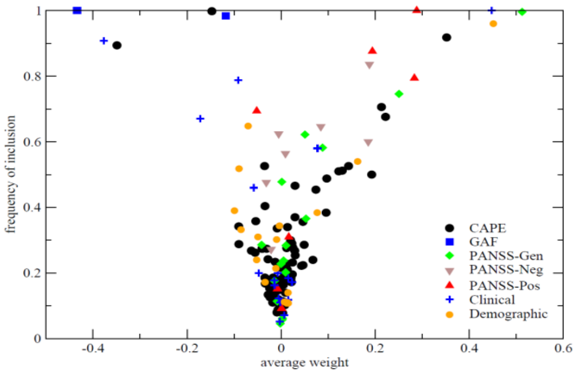

C. Symptomatic outcome T_6; PANSS, ill, demo, CANSAS

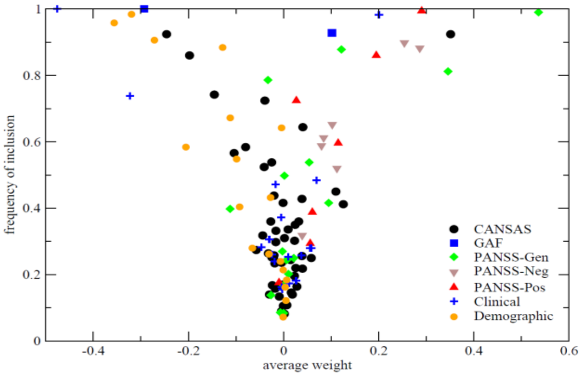

D. Symptomatic outcome T_6; PANSS, ill, demo, CAPE

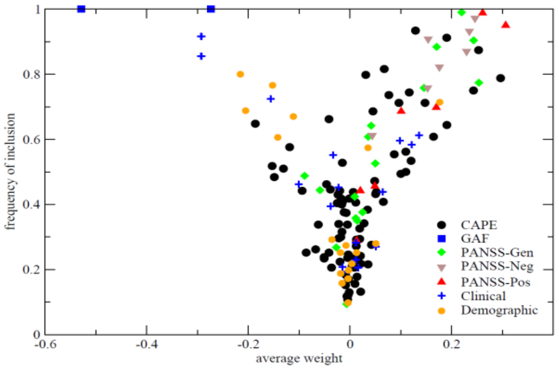

Frequency of inclusion of a feature against its (average) weight in the model; for symptomatic outcome (remission) models at T_3 (A and B) and T_6 (C and D), containing Positive and Negative Syndrome Scale (PANSS), demographic (demo), illness (ill) and need of care (CANSAS) related features (A and C) or PANSS, demographic, illness and lifetime psychotic experiences (CAPE) related features (B and D).

Supplementary Figure 4. Frequency of inclusion of a feature against its (average) weight in the model

A. Global outcome T_3; PANSS, ill, demo, CANSAS

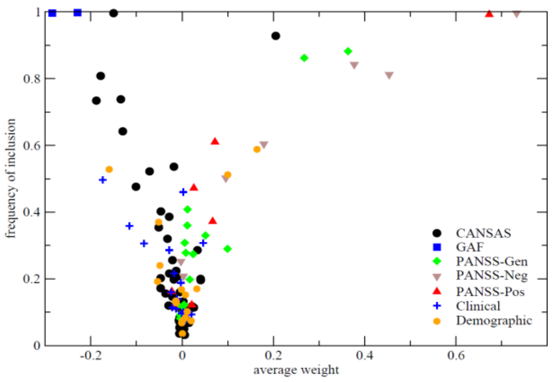

B. Global outcome T_3; PANSS, ill, demo, CAPE

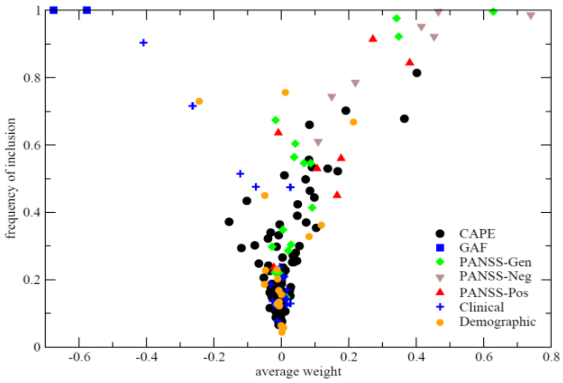

C. Global outcome T_6; PANSS, ill, demo, CANSAS

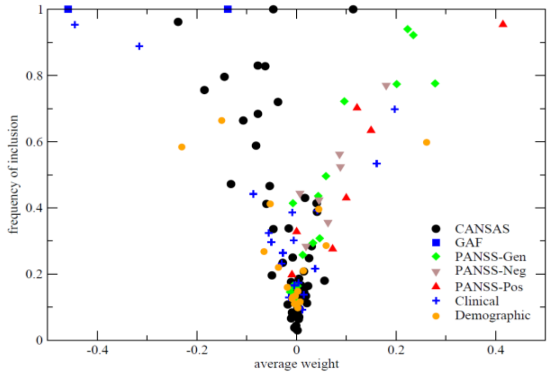

D. Global outcome T_6; PANSS, ill, demo, CAPE

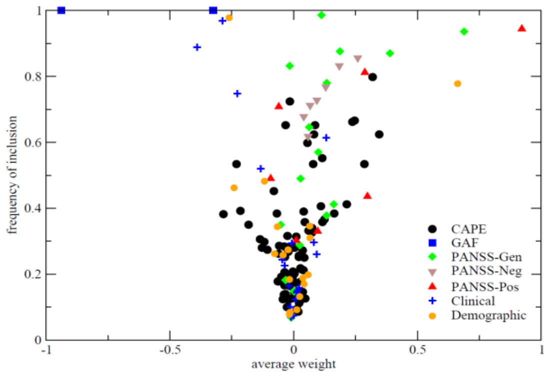

Frequency of inclusion of a feature against its (average) weight in the model; for global outcome models (GAF) at T_3 (A and B) and T_6 (C and D), containing Positive and Negative Syndrome Scale (PANSS), demographic (demo), illness (ill) and need of care (CANSAS) related features (A and C) or PANSS, demographic, illness and lifetime psychotic experiences (CAPE) related features (B and D).

Supplementary References

1 Andreasen, N. C., Flaum, M. & Arndt, S. The Comprehensive Assessment of Symptoms and History (CASH). An instrument for assessing diagnosis and psychopathology. *Arch. Gen. Psychiatry* **49**, 615-623 (1992).

2 Wing, J. K. *et al.* SCAN. Schedules for Clinical Assessment in Neuropsychiatry. *Arch. Gen. Psychiatry* **47**, 589-593 (1990).

3 Association, A. P. *Diagnostic and statistical manual of mental disorders (4th ed. text revision)*. 4th ed. text revision edn, (2000).

4 Development of the World Health Organization WHOQOL-BREF quality of life assessment. The WHOQOL Group. *Psychol. Med.* **28**, 551-558, doi:10.1017/s0033291798006667 (1998).

5 Kay, S. R., Fiszbein, A. & Opler, L. A. The positive and negative syndrome scale (PANSS) for schizophrenia. *Schizophr. Bull.* **13**, 261-276 (1987).

6 Kessler, R. & Ustun, T. The World Mental Health (WMH) survey initiative version of The World Health Organization (WHO) Composite International Diagnostic Interview (CIDI). *Int. J. Methods Psychiatr. Res.* **13**, 93-121, doi:10.1002/mpr.168 (2004).

7 Blyler, C. R., Gold, J. M., Iannone, V. N. & Buchanan, R. W. Short form of the WAIS-III for use with patients with schizophrenia. *Schizophr. Res.* **46**, 209-215 (2000).

8 Nuechterlein, K. H. & Dawson, M. E. Information processing and attentional functioning in the developmental course of schizophrenic disorders. *Schizophr. Bull.* **10**, 160-203 (1984).

9 Wohlberg, G. W. & Kornetsky, C. Sustained attention in remitted schizophrenics. *Arch. Gen. Psychiatry* **28**, 533-537, doi:10.1001/archpsyc.1973.01750340065011 (1973).

10 Bilder, R. M., Turkel, E., Lipschutz-Broch, L. & Lieberman, J. A. Antipsychotic medication effects on neuropsychological functions. *Psychopharmacol. Bull.* **28**, 353-366 (1992).

11 Brand, N. & Jolles, J. Learning and retrieval rate of words presented auditorily and visually. *J. Gen. Psychol.* **112**, 201-210, doi:10.1080/00221309.1985.9711004 (1985).

12 Benton, A. L., Sivan, A. B., de Hamsher, K. S., Varney, N. R. & Spreen, O. *Benton's Test of Facial Recognition*. (Oxford University Press, 1983).

13 van 't Wout, M., Aleman, A., Kessels, R. P., Larøi, F. & Kahn, R. S. Emotional processing in a non-clinical psychosis-prone sample. *Schizophr. Res.* **68**, 271-281, doi:10.1016/j.schres.2003.09.006 (2004).

14 Corcoran, R., Mercer, G. & Frith, C. D. Schizophrenia, symptomatology and social inference: investigating "theory of mind" in people with schizophrenia. *Schizophr. Res.* **17**, 5-13, doi:10.1016/0920-9964(95)00024-g (1995).

15 Cannon-Spoor, H. E., Potkin, S. G. & Wyatt, R. J. Measurement of premorbid adjustment in chronic schizophrenia. *Schizophr. Bull.* **8**, 470-484 (1982).

16 Phelan, M. *et al.* The Camberwell Assessment of Need: the validity and reliability of an instrument to assess the needs of people with severe mental illness. *Br. J. Psychiatry* **167**, 589-595 (1995).

17 Andresen, R., Caputi, P. & Oades, L. G. Interrater reliability of the Camberwell Assessment of Need Short Appraisal Schedule. *Aust. N. Z. J. Psychiatry* **34**, 856-861, doi:10.1080/j.1440-1614.2000.00814.x (2000).

18 Stefanis, N. C. *et al.* Evidence that three dimensions of psychosis have a distribution in the general population. *Psychol. Med.* **32**, 347-358 (2002).

19 Korver, N. *et al.* Genetic Risk and Outcome of Psychosis (GROUP), a multi-site longitudinal cohort study focused on gene-environment interaction: objectives, sample characteristics, recruitment and assessment methods. *Int. J. Methods Psychiatr. Res.* **21**, 205-221, doi:10.1002/mpr.1352 (2012).

20 Derks, E. M., Verweij, K. H., Kahn, R. S. & Cahn, W. C. The calculation of familial loading in schizophrenia. *Schizophr. Res.* **111**, 198-199, doi:10.1016/j.schres.2009.02.013 (2009).

21 McLaughlin, R. L. *et al.* Genetic correlation between amyotrophic lateral sclerosis and schizophrenia. *Nat Commun* **8**, 14774, doi:10.1038/ncomms14774 (2017).

22 Little, R. J. & Rubin, D. B. *Statistical Analysis with Missing Data*. (Wiley, 1987).

23 Schafer, J. L. Multiple imputation: a primer. *Stat. Methods Med. Res.* **8**, 3-15, doi:10.1177/096228029900800102 (1999).

24 caret: Classification and Regression Training (2018).

25 Kuhn, M. Caret package. *Journal of Statistical Software* **28**, 1-26 (2008).

26 Raamana, P. R. *neuropredict: easy machine learning and standardized predictive analysis of biomarkers*, 2017).
