# Supplementary material for: Individualized prediction of three- and six-year outcomes of psychosis in a longitudinal multicenter study: a machine learning approach
Source: NPJ Schizophr. 2021 Jul 2;7:34. doi: 10.1038/s41537-021-00162-3 (PMC8253813; doi:10.1038/s41537-021-00162-3)
Supplement: Supplementary file 2 — Reporting Summary [file 41537_2021_162_MOESM2_ESM.pdf]

nature research

Corresponding author(s): Hugo Schnack

Last updated by author(s): Jan 31, 2021

Reporting Summary

Nature Research wishes to improve the reproducibility of the work that we publish. This form provides structure for consistency and transparency in reporting. For further information on Nature Research policies, see our Editorial Policies and the Editorial Policy Checklist.

Statistics

For all statistical analyses, confirm that the following items are present in the figure legend, table legend, main text, or Methods section.

n/a	Confirmed	
☐	☒	The exact sample size (*n*) for each experimental group/condition, given as a discrete number and unit of measurement
☐	☒	A statement on whether measurements were taken from distinct samples or whether the same sample was measured repeatedly
☐	☒	The statistical test(s) used AND whether they are one- or two-sided<br>*Only common tests should be described solely by name; describe more complex techniques in the Methods section.*
☐	☒	A description of all covariates tested
☐	☒	A description of any assumptions or corrections, such as tests of normality and adjustment for multiple comparisons
☐	☒	A full description of the statistical parameters including central tendency (e.g. means) or other basic estimates (e.g. regression coefficient) AND variation (e.g. standard deviation) or associated estimates of uncertainty (e.g. confidence intervals)
☒	☐	For null hypothesis testing, the test statistic (e.g. *F*, *t*, *r*) with confidence intervals, effect sizes, degrees of freedom and *P* value noted<br>*Give P values as exact values whenever suitable.*
☒	☐	For Bayesian analysis, information on the choice of priors and Markov chain Monte Carlo settings
☐	☒	For hierarchical and complex designs, identification of the appropriate level for tests and full reporting of outcomes
☐	☒	Estimates of effect sizes (e.g. Cohen's *d*, Pearson's *r*), indicating how they were calculated

Our web collection on statistics for biologists contains articles on many of the points above.

Software and code

Policy information about availability of computer code

Data collection	no software was used to obtain the primary data of this study
Data analysis	R [version 3.4.0]. Packages used include the open source library for machine learning in R [v. 1.6.8], and the Caret package [v. 6.0.76]

For manuscripts utilizing custom algorithms or software that are central to the research but not yet described in published literature, software must be made available to editors and reviewers. We strongly encourage code deposition in a community repository (e.g. GitHub). See the Nature Research guidelines for submitting code & software for further information.

Data

Policy information about availability of data

All manuscripts must include a data availability statement. This statement should provide the following information, where applicable:

- Accession codes, unique identifiers, or web links for publicly available datasets
- A list of figures that have associated raw data
- A description of any restrictions on data availability

The data that support the findings of this study are available on request from the corresponding author. The data are not publicly available due to them containing information that could compromise research participant privacy or consent.

Field-specific reporting

Please select the one below that is the best fit for your research. If you are not sure, read the appropriate sections before making your selection.

☐ Life sciences ☒ Behavioural & social sciences ☐ Ecological, evolutionary & environmental sciences

For a reference copy of the document with all sections, see nature.com/documents/nr-reporting-summary-flat.pdf

Behavioural & social sciences study design

All studies must disclose on these points even when the disclosure is negative.

Study description

In the Genetic Risk and OUtcome in Psychosis (GROUP) prospective longitudinal cohort study, in- and out-patients with a psychotic disorder presenting consecutively at selected representative mental health services in representative geographical areas in the Netherlands and Belgium from January 8th, 2004 until February 6, 2008 were recruited. Inclusion criteria were: (1) psychotic disorder diagnosis according to the Diagnostic and Statistical Manual of Mental Disorders, Fourth Edition APA, (2) age 16-50 years (extremes included); (3) Dutch language proficiency; (4) ability to provide informed consent. Extensive genetic, cognitive, environmental, and outcome data were collected at baseline (T0), and three-year (T3) and six-year (T6) follow-up. The full GROUP sample at baseline included 1119 patients with variable illness duration, including recent onset psychosis. Here, we used data of 523 participants for whom outcome assessments at T3, and T6 were available, with a schizophrenia spectrum disorder (i.e. schizophrenia, schizophreniform disorder, schizoaffective disorder, delusional disorder, brief psychotic disorder, psychotic disorder: not otherwise specified), assessed with the Comprehensive Assessment of Symptoms and History or the Schedules for Clinical Assessment for Neuropsychiatry (See supplementary Figure S1 for selection process flow-chart). We assessed selection bias by comparing our sample on demographic and clinical characteristics to GROUP patients not included in this study.

The study protocol was approved by the Medical Ethical Review Board of the University Medical Centre Utrecht and by local review boards of participating institutes. Participants provided written informed consent.

For an elaborate description of the GROUP project, see its methods paper:
Korver, N. et al. Genetic Risk and Outcome of Psychosis (GROUP), a multi-site longitudinal cohort study focused on gene-environment interaction: objectives, sample characteristics, recruitment and assessment methods. Int. J. Methods Psychiatr. Res. 21, 205-221, doi:10.1002/mpr.1352 (2012).

Research sample

We included 523 patients with a schizophrenia spectrum disorder who had outcome assessments three (T3) and six (T6) years after baseline. Demographic and clinical baseline characteristics of the study sample and comparisons to patients excluded because of missing followup assessments are listed in Table 1 of the manuscript. Patients with unfavorable baseline characteristics were more likely to be lost to follow-up. At baseline, T3 and T6, 49%, 37% and 41% of patients were in symptomatic remission (according to the consensus definition by Andreasen et al. (2005)) respectively; 31%, 44% and 36% had good global functioning status (Global Assessment of Functioning (GAF) scale ≥65) at respective measurements. For symptomatic outcome, 65% and 64% of patients were stable at T3 and T6 relative to baseline, and 68% and 68% for global outcome.

The sample is thought to be representative of a relatively well-functioning subset of schizophrenia-spectrum disorder patients in need of specialist care.

This study employed the existing dataset from the GROUP project. Database release 5.0 was used in all analyses.

Rationale for study sample within GROUP: "The objectives of the GROUP study are two-fold: (1) investigating the genetic and environmental factors, and their interaction, contributing to the expression of psychosis; (2) investigating factors of vulnerability and protectiveness, and response to medication and clinical outcome." (Korver et al., 2012)

Sampling strategy

sampling: see above, convenience sample.

sample size calculation: in this study, sample size was determined by availability, that is based on the number of patient-participants within the GROUP study which had outcome measurements at three and six-year follow-up available.

The original GROUP sample size was determined as follows, and hinged on detecting differences between patients and their siblings; and siblings/patients and controls.

"In order to establish sufficient power to address the study objectives, a large sample for all cohorts is needed. In order to investigate gene–environment interaction in a case-only design (Khoury and Flandres, 1996), at least 1000 patients are required in order to establish a power of 89.7% (a = 0.05). A gene–environment study using a case-sibling design (Ottman, 1996) requires at least 1000 patients and 1000 siblings in order to reach a power of 81% (a = 0.05). To detect statistical differences (a = 0.05) between the cohorts longitudinally, at least 1000 patients, 1000 siblings, and 350 controls are needed to establish a power of 93% in a case–control and a case-sibling design (standard deviation = 0.15) on a continuous variable (cognitive function, schizotypy). For a sibling-control design, 1000 siblings and 350 controls would be required for a power of 89% (standard deviation = 0.10)." (Korver et al., 2006)

Data collection

see methods paper for the GROUP project by Korver et al. (2012)

Timing

from January 8th, 2004 until February 6, 2008 (inclusion for baseline).

Data exclusions

See below for data exclusions from the whole GROUP patient sample based on drop-out. exclusion criteria were pre-established.

Non-participation	drop-out: from 1119 patients participating in the GROUP project, n=498 did not have three and six-year follow-up (reasons stated: inability to track the patient, lack of motivation/time to participate or death). n=28 had missing or other diagnosis than schizophenia spectrum disorder or schizoaffective disorder. n=51 did not have any outcome data on symptomatic and global outcome.<br><br>response rate: Unfortunately, the response rate of the subjects approached was not evaluated.
Randomization	n.a.

Reporting for specific materials, systems and methods

We require information from authors about some types of materials, experimental systems and methods used in many studies. Here, indicate whether each material, system or method listed is relevant to your study. If you are not sure if a list item applies to your research, read the appropriate section before selecting a response.

Materials & experimental systems

n/a	Involved in the study
☒	☐ Antibodies
☒	☐ Eukaryotic cell lines
☒	☐ Palaeontology and archaeology
☒	☐ Animals and other organisms
☐	☒ Human research participants
☒	☐ Clinical data
☒	☐ Dual use research of concern

Methods

n/a	Involved in the study
☒	☐ ChIP-seq
☒	☐ Flow cytometry
☒	☐ MRI-based neuroimaging

Human research participants

Policy information about studies involving human research participants

Population characteristics	See above
Recruitment	See above<br><br>Self selection biases:<br>The GROUP study sample is known to represent a relatively well functioning subset of a population of schizophrenia-spectrum patients in need of specialist care. Generalization to other samples further might be hindered by exclusion of the most severely affected patients, either due to study drop-out, exclusion of patients with extensive missing data, or incompetence or unwillingness to give study consent. This is a limitation of our study mentioned in the manuscript.
Ethics oversight	Medical Ethical Review Board of the University Medical Centre Utrecht

Note that full information on the approval of the study protocol must also be provided in the manuscript.
